# A New Dimeric Copper(II) Complex of Hexyl Bis(pyrazolyl)acetate Ligand as an Efficient Catalyst for Allylic Oxidations

**DOI:** 10.3390/molecules26206271

**Published:** 2021-10-16

**Authors:** Luca Bagnarelli, Alessandro Dolmella, Carlo Santini, Riccardo Vallesi, Roberto Giacomantonio, Serena Gabrielli, Maura Pellei

**Affiliations:** 1 Chemistry Division, School of Science and Technology, University of Camerino, Via S. Agostino 1, 62032 Camerino, Italy; luca.bagnarelli@unicam.it (L.B.); carlo.santini@unicam.it (C.S.); riccardo.vallesi@unicam.it (R.V.); robert.giacomantonio@unicam.it (R.G.); 2 Department of Pharmaceutical and Pharmacological Sciences, University of Padova, Via Marzolo 5, 35131 Padova, Italy; alessandro.dolmella@unipd.it

**Keywords:** catalysis, copper, allylic oxidation, X-ray, spectroscopy

## Abstract

A new dimeric copper(II) bromide complex, [Cu(L^OHex^)Br(μ-Br)]_2_ (**1**), was prepared by a reaction of CuBr_2_ with the hexyl bis(pyrazol-1-yl)acetate ligand (L^OHex^) in acetonitrile solution and fully characterized in the solid state and in solution. The crystal structure of **1** was also determined: the complex is interlinked by two bridging bromide ligands and possesses terminal bromide ligands on each copper atom. The two pyrazolyl ligands in **1** coordinate with the nitrogen atoms to complete the Cu coordination sphere, resulting in a five-coordinated geometry—away from idealized trigonal bipyramidal and square pyramidal geometries—which can better be described as distorted square pyramidal, as measured by the τ and χ structural parameters. The pendant hexyloxy chain is disordered over two arrangements, with final site occupancies refined to 0.705 and 0.295. The newly synthesized complex was evaluated as a catalyst in copper-catalyzed C–H oxidation for allylic functionalization through a Kharasch–Sosnovsky reaction without any external reducing agent. Using 0.5 mol% of this catalyst, and *tert-*butyl peroxybenzoate (Luperox) as an oxidant, allylic benzoates were obtained with up to 90% yield. The general reaction time was only slightly decreased to 24 h but a very significant decrease in the alkene:Luperox ratio to 3:1 was achieved. These factors show relevant improvements with respect to classical Kharasch–Sosnovsky reactions in terms of rate and amount of reagents. The present study highlights the potential of copper(II) complexes containing functionalized bis(pyrazol-1-yl)acetate ligands as efficient catalysts for allylic oxidations.

## 1. Introduction

**Scheme 1:**
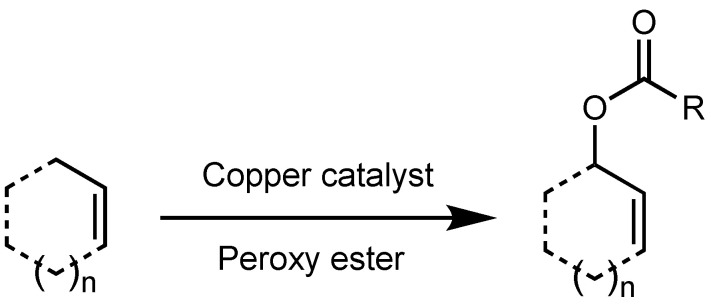
Kharasch–Sosnovsky reaction catalyzed by copper catalysts: copper(I) salts/complexes or copper(II) species requiring the use of an external reducing agent such as phenylhydrazine.

Nowadays, the focus on innovative and proper chemical transformations using catalytic metal complexes to devise functionalized intermediates remains indispensable in many chemical areas. The allylic oxidation of alkenes [1,2,3,4] allows access to highly functionalized compounds with a high added value, such as alcohols, aldehydes, ketones, epoxides, and carboxylic acids, which are suitable for further manipulations in synthetic and industrial applications [5,6]. These chemical derivatives can be generally obtained throughout an epoxidation and a dihydroxylation reaction, but, in contrast, allylic oxidation with copper catalysts and peroxy ester oxidant, often referred to as the Kharasch–Sosnovsky reaction, generates products with the olefin left intact [7,8,9,10,11,12,13] (Scheme 1). In this way, among the several transformations existing for olefins, allylic oxidation has demonstrated a very good aptitude for synthetic purposes that complement epoxidation and dihydroxylation. Although it is a powerful method that has been extensively studied, its use in synthesis has been limited because of the need for a long reaction time and superstoichiometric amounts of olefin (2.5–10 equivalents) [14]. Classically, the Kharasch–Sosnovsky reaction employs simple Cu(I) salts [10,15,16,17,18,19], able to promote the homolysis of the peroxy ester. The exact reaction mechanism is quite complex, even if no clear relationship has been found between the use of a particular ligand and copper salts [8,10,20,21]. In recent years, the search for improved Cu-based catalysts to promote allylic oxidation and widen the related field of applications has been an important topic in the scientific community [22]. In particular, the copper-catalyzed allylic oxidation of olefins is the key step in the synthesis of many natural products and pharmaceuticals [23,24,25,26,27,28,29]. The ease of handling and the stability of copper(II) salts make them valuable and suitable catalysts [8,10,20,30,31,32,33]. However, in the Kharasch–Sosnovsky reaction, the use of Cu(I) complexes results in better and more reproducible yields compared to Cu(II) species, which, in addition, require the use of external reducing agents. The mechanism of the reaction has been controversial, and different alternatives have been presented over the years [34,35,36,37,38,39]. This can be explained by how copper chemistry is incredibly diverse, and, depending on its oxidation state, this metal can powerfully catalyze reactions involving one- and/or two-electron mechanisms.

Complexes containing κ^3^N,N,O-heteroscorpionate ligands, derived from bis(pyrazol-1-yl)acetates [40,41,42], are of particular interest due to their coordination behavior in organometallic chemistry [42,43,44], both as metalloenzyme models [42,45,46,47,48] and as starting reagents in the synthesis of bifunctional ligands and related metal complexes which are useful for their biological activity [49,50,51,52,53,54]. Furthermore, in the last years, interest in the catalytic activity of this type of metal complex has grown considerably [55]. Currently, although few catalytic studies have been carried out, some of them are active catalysts in processes such as olefin polymerization [56,57,58,59] and oxidation [60], C–H bond functionalization/activation [61], ring-closing metathesis [62], and the reduction of protons from organic acids or aqueous solutions [63]. In addition, bis(pyrazol-1-yl)acetates are suitable for the preparation of solid-phase-grafted ligands [64,65,66] and in the development of hybrid organic–inorganic materials [67], which are not only of interest due to the advantages of supported catalysts in potential applications [68,69] but also to favor the formation of mono-ligand complexes without the need for sterically demanding substituents [69,70].

In the last few years, there has been increased interest in copper-catalyzed organic reactions [8,22,71,72], as copper is an earth-abundant metal, making its use more cost-effective and sustainable than precious transition metal catalysts. The most popular species employed in the Kharasch–Sosnovsky oxidation are the Cu(I) or Cu(II) complexes bearing oxazoline-based ligands [73]. The use of additives that accelerate the rate of this reaction has been constant. One of the most important additives used in these reactions is phenylhydrazine as a reducing agent of Cu(II) species to Cu(I) [30].

Recently, inspired by this growth of studies, we successfully tested the catalytic activity of novel copper(II) complexes containing the ester derivatives of bis(azol-1-yl)acetate ligands by the Kharasch–Sosnovsky reaction [74]. Thus, the present study reports the syntheses of a new dimeric copper(II) bromide complex, [Cu(L^OHex^)Br(μ-Br)]_2_ (**1**), containing the hexyl bis(pyrazol-1-yl)acetate ligand useful as a catalyst in copper-catalyzed C–H oxidation for allylic functionalization. In this way, we emphasize the use of a Cu(II) catalyst in Kharasch–Sosnovsky reactions without the need for superstoichiometric amounts of olefin and external reducing agents, such as phenylhydrazine, considered toxic by the United Nations Environment Program [75], decreasing the generation of waste and preventing the use of unnecessary reagents.

## 2. Results and Discussion

### 2.1. Synthesis and Characterization

**Scheme 2:**
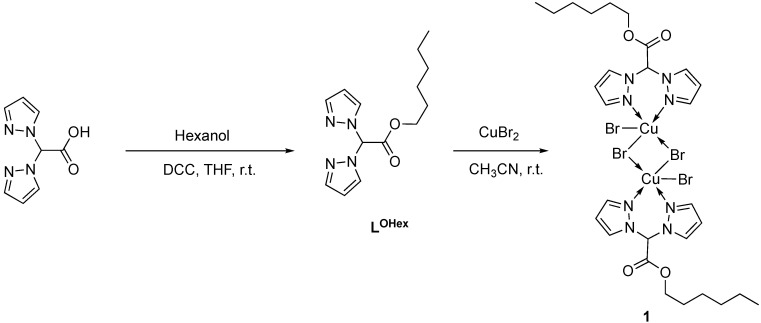
Synthesis of ligand L^OHex^ and complex **1**.

The ligand L^OHex^ was prepared according to the procedure reported in Scheme 2, using HC(COOH)(pz)_2_ and 1-hexanol as starting materials. L^OHex^ is soluble in common organic solvents and slightly soluble in water. The ^1^H- and ^13^C-NMR spectra of the ligand, recorded in CDCl_3_, CD_3_CN, and DMSO-d_6_ solutions, showed all the expected signals and, due to magnetic equivalence, only one set of resonances for the pyrazole rings. The molecular structure of L^OHex^ is confirmed by the presence of the peaks at *m*/*z* 277 and 299 of the [L^OHex^ + H]^+^ and [L^OHex^ + Na]^+^ species, respectively, in the positive-ion spectrum in CH_3_CN solution.

The copper complex [Cu(L^OHex^)Br(μ-Br)]_2_ (**1**, Scheme 2) was prepared from the reaction of L^OHex^ with CuBr_2_ in acetonitrile suspension at room temperature. Complex **1** is soluble in methanol, acetonitrile, chloroform, dimethyl sulfoxide, and acetone; it was completely characterized in the solid state and in solution. In particular, complex **1** shows an intense absorption at 1740 cm^−1^ due to the asymmetric stretching of the C=O groups. No significant variation with respect to the free ligand (*v*_asym_ C=O, 1754 cm^−1^) has been observed, confirming that in the solid state the carbonyl groups are not involved in the coordination of copper, in accordance with the X-ray crystal structure with the ligands chelating in a κ^2^N,N’ bidentate fashion. In the ESI-MS(+) spectrum of **1**, the peaks at *m*/*z* of 339 and 615, due to the [L^OHex^ − H + Cu]^+^ and [2L^OHex^ − H + Cu]^+^ species, respectively, confirm the complex formation.

### 2.2. Investigation on the Catalytic Activity of Complex **1** in the Allylic Oxidation of Alkenes

The high catalytic activity of copper complexes found during our recent work [74] stimulated us to develop a new copper(II) complex (**1**) in order to study its catalytic potential towards the oxidation of alkenes. In fact, we started from the results [74] obtained during our previous experimental tests with analogous copper(II) complexes—such as the [HC(COOH)(pz^Me2^)_2_] (L^2OHex^) derivatives [(L^2OHex^)CuCl_2_] and [(L^2OHex^)CuBr_2_]—in which we obtained an 85% yield, using 5 mol% of [(L^2OHex^)CuBr_2_], with a 5:1 ratio of alkenes and Luperox, performing the reaction at 60 °C over 6 h. Herein, we developed a further strategy for allylic oxidation in order to reduce the amount of catalyst and increase the yield. In particular, the new L^OHex^ derivative **1** was chosen to enlarge the chemical pattern of copper complexes, allowing for the formation of these important products. To determine the catalytic activity of complex **1**, a series of preliminary tests were carried out.

The starting point was related to the use of 5 mol% of the catalyst **1**—leaving the reaction time at 6 h and the temperature at 60 °C—focused on the oxidation of cyclohexene **2** using *t*-butyl perbenzoate **3** (Luperox) as a reactant to the corresponding ester **4** with a **2**:**3** ratio of 5:1, achieving only 73% yield (Table 1, entry *1a*). After this first trial, we made several attempts to define the mutual activity of complex **1**. The best result was in fact observed using 0.5 mol% of **1**, a slight excess of cyclohexene (**2**:**3** ratio = 3:1), at 60 °C (Table 1, entry *1h*) for 24 h.

Complex **1** showed very virtuous catalytic performance. Using a **2**:**3** ratio of 5:1, with 5 mol% of the catalyst over 24 h, the yield was almost quantitative (Table 1, entry *1b*). This yield decreased a bit upon reducing the ratio of **2**:**3** to 3:1, due to the scarce and non-proportional amount of cyclohexene **2** and Luperox **3** (Table 1, entry *1c*). A noteworthy upgrade was obtained by carrying out the reaction with 1 mol% of **1**, for 24 h at 60 °C; in this setting, the yields were comparable with those obtained before (Table 1, entry *1d* of 77% and Table 1, entry *1e* of 74%). So, based on the above-mentioned optimization steps, the catalytic activity of complex **1** has been further developed, decreasing the amount to 0.5 mol%. Nonetheless, an almost quantitative yield can be reached by increasing the **2**:**3** ratio to 10:1, using 0.5 mol% of the catalyst, and obtaining 95% yield after 24 h at 60 °C. A reduction of the **2**:**3** ratio means a reduction in the amount of alkene that could be, in some way, precious and not easily available, thus reducing the waste of the starting materials. Therefore, the use of a 3 to 1 ratio of cyclohexene **2** and Luperox **3** allowed us to obtain a very good yield, up to 90%, under the same reaction conditions (Table 1, entry *1h*).

**Figure 1:**
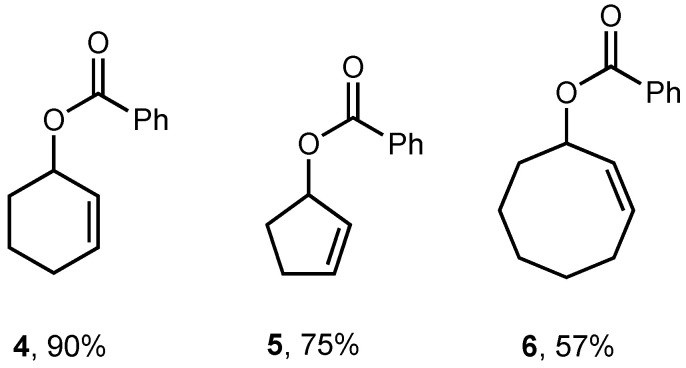
Allyl derivatives **4**–**6** obtained using 0.5 mol% of complex **1**.

Finally, once we confirmed the catalytic activity of this complex, we examined the feasibility of this reaction on cyclopentene and cyclooctene, obtaining, in both cases, good yields, under the same experimental conditions (Figure 1). 

It is important to note that the presence of a hexyl group in the ester moiety appears to have a positive effect on the reaction yields. In addition, the chelating properties of the ligand [HC(COOH)(pz)_2_] (L^OHex^) allows for the formation of the dimeric compound [Cu(L^OHex^)Br(μ-Br)]_2_, where the second copper(II) ion might participate in the catalytic process, increasing the yields by using 0.5 mol% of complex **1**. Moreover, based on the chemical structure of alkenes, the yields are variable; in fact, considering the conformational structure of the alkenes, the yield increased in the case of cyclohexene, while a lower interaction with the catalyst resulted in a lower yield for cyclopentene and cyclooctene.

In this paper, we have tested the catalytic properties of **1** on cyclohexene, cyclopentene, and cyclooctene. In fact, the substrate nature has been somewhat limited and most of the work in the literature has been performed with hydrocarbons, mainly cyclic alkenes or very simple linear alkenes. Cyclohexene has been the substrate of choice in most studies, usually providing the highest yields among related olefins under a wide range of conditions. Cyclooctene also proceeds with moderate to good yields. In contrast, the behavior of cyclopentene and cycloheptene is less predictable. In Table 2 we have reported, for comparison, conditions used and results obtained in recent years using other Cu(I) or Cu(II) salts or complexes as catalysts for allylic oxidations of simple cyclic alkenes.

### 2.3. X-ray Crystallography

**Figure 2:**
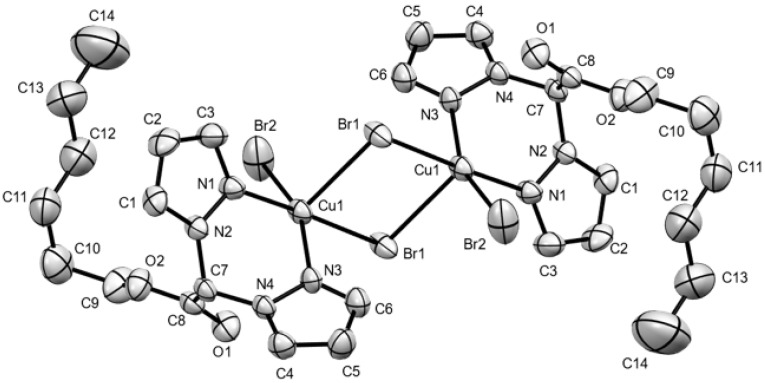
ORTEP-like molecular structure of [Cu(L^OHex^)Br(μ-Br)]_2_ (**1**), with thermal ellipsoids drawn at the 30% probability level. Disordered molecular fragments and hydrogen atoms were removed for clarity. The labeling of the atoms in the two (identical) halves of the dimeric complex is the same.

**Figure 3:**
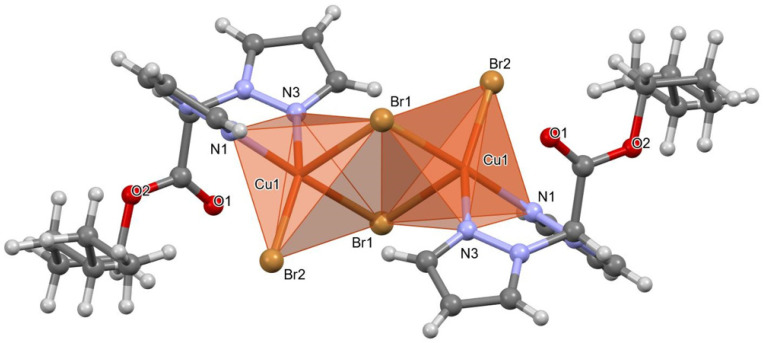
Mercury [90] ball-and-stick representation of the dimeric complex, highlighting the square pyramidal environment of the copper atoms. For clarity, only one of the hexyloxy chain arrangements is shown. Monomeric units on the left and on the right show, respectively, the apical bromide and basal square planes.

A summary of the crystal/structure refinement data is given in Table S1 (Supplementary Materials), and selected bond lengths and angles are reported in Table 3. An ORTEP-like [80] representation of the complex is given in Figure 2; Figure 3 highlights the distorted square pyramidal polyhedra of the two Cu centers. The crystal structure investigation revealed that, in the solid state, the compound exists as a dimer of formula [Cu(L^OHex^)Br(μ-Br)]_2_, with the Br(1) ions binding two symmetry-related units to each other. To the best of our knowledge, this complex is one of the few mono- or di-nuclear bis-pyrazolyl acetate copper complexes [81,82] with uncoordinated acetate moieties, and also one of the relatively not-so-abundant copper complexes showing µ-bridging bromide ions coupled with two pentacyclic N-based ligands [83,84,85,86,87,88], described in the CCDC repository [89].

In the dimer, two µ-bridging Br(1) and two Cu atoms define a Cu_2_Br_2_ tetracycle, while, upon coordination, the bis-pyrazolyl ligand makes with the copper atom a six-membered cycle puckered in a *boat* shape. In the latter, the four nitrogen atoms N(1)/N(4) lie in the same plane, whereas C(7) and Cu(1) atoms are at the ‘stern’ and ‘prow’ positions. The mean planes encompassing the five-membered pyrazolyl rings N(1):C(3) and N(3):C(6) make dihedral angles of 27.1 and 33.3°, respectively, with the N(1)/N(4) plane, and also make an angle of 59.8° with each other. Atoms C(9) to C(14) of the hexyl ester are disordered in two alternate arrangements, which have been conveniently modeled by means of SHELXL restraints [91]. The aliphatic chain is placed in such a way as to fold towards the N(1):C(3) ring, with C(12)/C(12A) approximately 4 Å apart from the ring centroid. The Cu atom is penta-coordinated and the environment has a distorted square planar shape; this seems to be the preferred shape in similar compounds [82,83,84,85,86,87,88]. Sitting in the pyramid basal plane are the N(1) and N(3) atoms of the two pyrazolyl rings, which are in *trans* position with the Br(1) and Br(2) atoms, respectively. The apical position is taken by a symmetry-related bridging Br(1)^I^ atom (at 1−*x*, 1−*y*, 1−*z*). The departure from the ideal arrangement is measured by the τ and χ parameters (0.32 and 0.36, respectively) [92,93] and by the bond angle values listed in Table 3, all reasonably close to the ideal values of 90 and 180°, except for the N(3)−Cu(1)−Br(2) angle of 157.34 (7)°.

As for metal-involving bonds, the Cu(1)−Br(2) length of the terminal bromide ion (see Table 3) is about 0.06 Å shorter than the Cu(1)−Br(1) distance (2.3687 (6) vs. 2.4302 (4) Å); the Cu(1)−Br(1)^I^ bond length of the µ-bridging Br ion is instead appreciably longer at 2.7600 (5), about 0.33 Å longer than the terminal Cu−Br bond. This value is higher than the reported average for similar compounds (2.59 Å) but fits within the reported range (2.37–3.06 Å) [82,83,84,85,86,87,88]. Similar considerations can apply to the Cu−N(1) and Cu−N(3) distances: respectively, 2.017 (2) and 2.042 (3) Å (mean: 2.00, range: 1.97–2.09 Å). The situation closely matches that found in the two known compounds that also show a bis-pyrazolyl moiety [82,86]. The N–N and C–N bond distances in the bis-pyrazolyl residues, and the C−C bonds in the hexyl chain, appear in line with known data and do not deserve further comment. It is instead worth noting that the O(1) oxygen of the carboxylic moiety is roughly in *trans* position with respect to the symmetry-related bridging Br(1) atom (angle O(1)−Cu(1)−Br(1)^I^ of 162.6°), in a virtual sixth Cu coordination position; however, the Cu(1)-O(1) distance is 3.174 Å, well above the sum of the Cu and O vdW radii (1.92 Å). The same situation was found in a recent report [82]. 

The crystal packing diagram [90] of **1** (Figure S3, Supplementary Materials) shows no strong intermolecular contacts. A few loose intermolecular contacts are established by C(7), C(8), H(1), and H(7) atoms with the Br(1) atom. In Table S2 (Supplementary Materials) we indicate these as having an interatomic distance about 0.1 Å smaller than the sum of the pertaining vdW radii. These contacts propagate both in the directions of the *b*- and *c*-axes, yielding a bi-dimensional network running along to the *bc* plane and containing all the Cu_2_Br_2_ units. Above and below this plane, we find two layers containing the aliphatic hexyl chains, thus defining a ‘sandwich’ structure with the layer containing the copper and bromine atoms. This motif is repeated along the *a*-axis. No π–π interactions appear to involve the pyrazolyl rings.

## 3. Experimental Section

### 3.1. Materials and Instruments

All syntheses and handling were carried out under a dry and oxygen-free atmosphere, using standard Schlenk techniques. All solvents were dried, degassed, and distilled prior to use. Elemental analyses (C,H,N,S) were performed with a Fisons Instruments EA-1108 CHNS-O Elemental Analyzer (Thermo Fisher Scientific Inc., Waltham, MA, USA). Melting points were taken on an SMP3 Stuart Scientific Instrument (Bibby Sterilin Ltd., London, UK). IR spectra were recorded from 4000 to 700 cm^−1^ on a PerkinElmer Frontier FT-IR instrument (PerkinElmer Inc., Waltham, MA, USA), equipped with a single-reflection universal diamond ATR top-plate. IR annotations used: m = medium, s = strong, sh = shoulder, vs = very strong, vw = very weak, w = weak. ^1^H- and ^13^C-NMR spectra were recorded with an Oxford AS400 Varian Spectrometer (400.4 MHz for ^1^H and 100.1 MHz for ^13^C) (Agilent Technologies Inc, Santa Clara, CA, USA) or with a 500Bruker Ascend (500.1 MHz for ^1^H and 125 MHz for ^13^C) (Bruker BioSpin Corporation, 15 Fortune Drive, Billerica, MA, USA). Referencing was relative to tetramethylsilane (TMS) (^1^H and ^13^C). NMR annotations used were as follows: d = doublet, m = multiplet, s = singlet, t = triplet. Electrospray ionization mass spectra (ESI-MS) were obtained in positive- (ESI-MS(+)) or negative-ion (ESI-MS(−)) mode on an Agilent Technologies Series 1100 LC/MSD Mass Spectrometer (Agilent Technologies Inc., Santa Clara, CA, USA), using a water or acetonitrile mobile phase. The compounds were added to reagent grade water or acetonitrile to give approximately 0.1 mM solutions, injected (1 μL) into the spectrometer via a Hewlett Packard 1090 Series II UV-Visible HPLC system (Agilent Technologies Inc., Santa Clara, CA, USA) fitted with an autosampler. The pump delivered the solutions to the mass spectrometer source at a flow rate of 300 mL/min, and nitrogen was employed both as a drying and nebulizing gas. Capillary voltages were typically 4000 V and 3500 V for the ESI-MS(+) and ESI-MS(−) mode, respectively. Confirmation of all major species in this ESI-MS study was supported by a comparison of the observed and predicted isotope distribution patterns, the latter calculated using the IsoPro 3.1 computer program (T-Tech Inc., Norcross, GA, USA). Gas chromatography-mass spectra (GC-MS) analyses were obtained on an Agilent GC(6850N)/MS(5973N) (Stevens Creek Blvd, Santa Clara, CA, USA); electronic impact technique (70 eV); GC/MSD software; HP-5MS column (30 m, Id 0.25 µm, film thickness 0.25 µm).

### 3.2. Synthesis

All reagents were purchased from Merck KGaA (Merck Life Science S.R.L., Via Monte Rosa, 93, Milano, Italy) and used without further purification. The ligand HC(COOH)(pz)_2_ was prepared by the literature methods [44]; HC(COOHex)(pz)_2_ (L^OHex^) was prepared by changing the literature procedure [74].

#### 3.2.1. Synthesis of HC(COOHex)(pz)_2_ (L^OHex^)

In a round-bottom flask, the ligand HC(COOH)(pz)_2_ (0.769 g, 4.000 mmol) and the 1-hexanol (HexOH, 0.409 g, 4.000 mmol) were added to tetrahydrofuran (THF, 50 mL), obtaining a suspension left under magnetic stirring and cooled at 0 °C. Subsequently, a solution of N,N′-dicyclohexylcarbodiimide (DCC, 1.651 g, 8.000 mmol) in THF (50 mL) was added dropwise; the mixture was stirred for 24 h at room temperature. The THF was evaporated at reduced pressure and ethyl acetate (EtOAc) was poured into the round-bottom flask. The obtained mixture was filtered and the mother liquors were washed with a citric acid solution (pH~3, 2 × 50 mL) and a saturated NaHCO_3_ solution (2 × 50 mL) and dried with Na_2_SO_4_. The mixture was filtered, and EtOAc was evaporated under reduced pressure. The residue was purified by a chromatographic column (SiO_2_; elution with cyclohexane:EtOAc 90:10 and then cyclohexane:EtOAc 80:20) and dried under reduced pressure, obtaining L^OHex^ as a pale yellow oily product, with a yield of 80%. ^1^H-NMR (CDCl_3_, 293 K): δ 0.88 (t, 3H, O(CH_2_)_5_C*H*_3_), 1.25–1.31 (m, 6H, O(CH_2_)_2_(C*H*_2_)_3_CH_3_), 1.61–1.68 (m, 2H, OCH_2_C*H*_2_(CH_2_)_3_CH_3_), 4.29 (t, 2H, OC*H*_2_(CH_2_)_4_CH_3_), 6.36 (t, 2H, 4-C*H*), 7.09 (s, 1H, C*H*COO), 7.61 (d, 2H, 5-C*H*), 7.77 (d, 2H, 3-C*H*). ^1^H-NMR (CD_3_CN 293 K): δ 0.89 (t, 3H, O(CH_2_)_5_C*H*_3_), 1.24–1.31 (m, 6H, O(CH_2_)_2_(C*H*_2_)_3_CH_3_), 1.55–1.60 (m, 2H, OCH_2_C*H*_2_(CH_2_)_3_CH_3_), 4.23 (t, 2H, OC*H*_2_(CH_2_)_4_CH_3_), 6.36 (t, 2H, 4-C*H*), 7.28 (s, 1H, C*H*COO), 7.57 (d, 2H, 5-C*H*), 7.84 (d, 2H, 3-C*H*). ^1^H-NMR (DMSO-d_6_ 293 K): δ 0.81 (t, 3H, O(CH_2_)_5_C*H*_3_), 1.13–1.19 (m, 6H, O(CH_2_)_2_(C*H*_2_)_3_CH_3_), 1.47 (mbr, 2H, OCH_2_C*H*_2_(CH_2_)_3_CH_3_), 4.14 (t, 2H, OC*H*_2_(CH_2_)_4_CH_3_), 6.33 (t, 2H, 4-C*H*), 7.56 (d, 2H, 5-C*H*), 7.71 (s, 1H, C*H*COO), 7.94 (d, 2H, 3-C*H*). ^13^C-NMR (CDCl_3_, 293 K): δ 13.9 (O(CH_2_)_5_*C*H_3_); 22.4, 25.2, 28.2, 31.2 (OCH_2_(*C*H_2_)_4_CH_3_); 67.2 (O*C*H_2_(CH_2_)_4_CH_3_); 74.6 (*C*HCOO); 107.3 (4-*C*_Pz_); 130.1 (5-*C*_Pz_); 140.9 (3-*C*_Pz_); 164.4 (*C*O). IR (cm^−1^): 3322 vw, 3139 w, 3109 w, 2956 m, 2923 s, 2871 m, 2851 m (C–H); 1754 vs (*v*_asym_ C=O); 1626 w, 1574 w, 1517 m (C=C/C=N); 1465 m, 1451 m, 1436 m, 1393 vs, 1385 vs, 1354 m, 1329 m, 1292 vs, 1253 vs, 1223 vs, 1213 vs, 1187 m, 1161 vs, 1088 vs, 1053 vs, 1022 m, 993 m, 972 s, 957 vs, 938 sh, 912 s, 905 vs, 892 m, 858 m, 845 m, 827 m, 806 vs, 777 vs, 762 vs, 725 s. ESI-MS(+) (major positive ions, CH_3_CN), *m*/*z* (%): 209 (10) [L^OHex^ − pz]^+^, 277 (15) [L^OHex^ + H]^+^, 299 (100) [L^OHex^ + Na]^+^. ESI-MS(−) (major negative ions, CH_3_CN), *m*/*z* (%): 147 (100) [HC(pz)_2_]^−^. Elemental analysis (%): calculated for C_14_H_20_N_4_O_2_: C, 60.85; H, 7.30; N, 20.28; found: C, 60.50; H, 7.07; N, 19.80.

#### 3.2.2. Synthesis of [Cu(L^OHex^)Br(μ-Br)]_2_ (**1**)

In a round-bottom flask, the ligand L^OHex^ (0.277 g, 1.0 mmol) was dissolved in CH_3_CN (50 mL). Then, a CH_3_CN solution (50 mL) of CuBr_2_ (0.223 g, 1.0 mmol) was added, and the resulting red-violet solution was stirred at room temperature for 18 h. At the end of the reaction, the solution was filtered and the brown-violet complex [Cu(L^OHex^)Br(μ-Br)]_2_, dried at reduced pressure, was obtained at a 90% yield. 

Single crystals of [Cu(L^OHex^)Br(μ-Br)]_2_, suitable for X-ray analysis, were obtained by the slow evaporation of an acetone solution of **1**. Melting point: 163–166 °C. IR (cm^−1^): 3146 w, 3134 w, 3122 w, 3101 m, 2983 w, 2952 m, 2929 m, 2908 sh, 2865 m (C–H); 1740 vs (ν_asym_ C=O); 1626 w, 1525 sh, 1515 m, 1463 sh, 1455 m (C=C/C=N); 1402 s, 1373 w, 1356 w, 1345 w, 1286 vs, 1255 s, 1227 vs, 1195 s, 1179 m, 1102 m, 1094 m, 1068 m, 1058 vs, 1019 w, 989 m, 976 m, 951 m, 922 m, 911 m, 878 w, 864 m, 823 m, 803 m, 762 vs, 723 m. ESI-MS(+) (major positive ions, CH_3_CN), *m*/*z* (%): 145 (90) [CuBr]^+^, 299 (10) [L^OHex^ + Na]^+^, 339 (100) [L^OHex^ − H + Cu]^+^, 615 (25) [2L^OHex^ − H + Cu]^+^. ESI-MS(−) (major negative ions, CH_3_OH), *m*/*z* (%): 222 (100) [CuBr_3_]^−^. Elemental analysis (%) calculated for C_14_H_20_Br_2_CuN_4_O_2_: C, 33.65; H, 4.03; N, 11.21; found: C, 33.85; H, 4.00; N, 11.01.

#### 3.2.3. General Procedure for the Synthesis of Compounds **4**–**6**

The reactions were performed in a sealed vial in which 0.5 mol% of complex **1**, 1 mmol of *t-*butyl peroxybenzoate (Luperox), and, finally, 3 mmols of the appropriate alkene were introduced, and the reaction was stirred at 60 °C for 24 h. The workup consisted of a pre-pad chromatography column (SiO_2_, elution with 150 mL of *n*-hexane:EtOAc 70:30). The purification of the crude residue was performed by a chromatography column (SiO_2_, elution with *n*-hexane:EtOAc 98:2) to give pure products.

##### Synthesis of **4**

Yield 90%. Colourless oil. IR (cm^−1^): 3064 w, 3033 w, 2934 m, 2868 w, 2835 w (C–H); 1710 vs (ν_asym_ C=O); 1602 m, 1585 m, 1491 w, 1451 s, 1396 m, 1336 m, 1314 s, 1266 vs, 1176 s, 1163 s, 1143 m, 1109 vs, 1100 sh, 1069 vs, 1057 vs, 1051 vs, 1026 vs, 1008 vs, 1001 vs, 915 vs, 853 m, 837 m, 806 m, 708 vs. ^1^H-NMR (CDCl_3_, 293 K): δ 1.70–1.77 (m, 1H, CH_2_C*H*_2_CH_2_CH=CH), 1.83–2.21 (m, 5H, CHCO(C*H*_2_)_3_), 5.52–5.55 (m, 1H, CH_2_C*H*=CH), 5.84–5.87 (m, 1H, CH=C*H*CHCO), 6.01–6.05 (m, 1H, C*H*CO), 7.45 (t, 2H, *J* = 7.5 Hz, C_6_*H*_5_), 7.57 (t, 1H, *J* = 7.5 Hz, C_6_*H*_5_), 8.08 (d, 2H, *J* = 10 Hz, C_6_*H*_5_). ^13^C-NMR (CDCl_3_, 293 K): δ 19.0, 25.0, 28.4, 68.6, 125.8, 128.3, 129.6, 130.8, 132.7, 132.8, 166.2. GC-MS (70eV), *m/z*: 202 ([M^+^], 11), 157 (4), 120 (3), 105 (100), 97 (13), 81 (30), 77 (38), 51 (13), 41 (8). Elemental analysis (%) calculated for C_13_H_14_O_2_: C, 77.20; H, 6.98; found: C, 77.96; H, 7.14.

##### Synthesis of **5**

Yield 75%. Pale yellow oil. IR (cm^−1^): 3062 w, 2973 w, 2939 w, 2855 w (C–H); 1709 vs (ν_asym_ C=O); 1602 m, 1585 m, 1491 w, 1451 s, 1366 m, 1338 vs, 1314 s, 1267 vs, 1176 s, 1161 m, 1110 vs, 1100 vs, 1069 vs, 1026 vs, 1001 s, 946 vs, 937 vs, 917 s, 884 vs, 850 m, 806 m, 788 m, 708 vs. ^1^H-NMR (CDCl_3_, 293 K): δ 1.96–2.03 (m, 1H, CH=CHC*H*_2_), 2.38–2.47 (m, 2H, C*H*_2_CHCO and CH=CHC*H*_2_), 2.55–2.66 (m, 1H, C*H*_2_CHCO), 5.96–5.99 (m, 2H, CH_2_C*H*=C*H*), 6.17–6.19 (m, 1H, C*H*CO), 7.45 (t, 2H, *J* = 7.5 Hz, C_6_*H*_5_), 7.56 (t, 1H, *J* = 7.5 Hz, C_6_*H*_5_), 8.05–8.07 (m, 2H, C_6_*H*_5_). ^13^C-NMR (CDCl_3_, 293 K): δ 29.9, 31.2, 81.1, 128.3, 129.4, 129.6, 130.7, 132.7, 137.7, 166.6. GC-MS (70 eV), *m*/*z*: 188 ([M^+^], 26), 170 (7), 143 (10), 122 (5), 105 (100), 84 (55), 77 (56), 67 (97), 51 (26), 41 (18), 39 (20), 29 (5). Elemental analysis (%) calculated for C_12_H_12_O_2_: C, 76.57; H, 6.43; found: C, 76.09; H, 6.05.

##### Synthesis of **6**

Yield 57%. Colourless oil. IR (cm^−1^): 3063 vw, 3023 w, 2927 m, 2856 m (C–H); 1714 vs (ν_asym_ C=O); 1602 w, 1585 w, 1491 w, 1451 s, 1362 w, 1314 s, 1270 vs, 1195 m, 1176 m, 1150 m, 1110 vs, 1069 vs, 1025 vs, 1003 sh, 947 vs, 893 m, 868 w, 851 w, 835 w, 805 w, 781 m, 754 vs, 708 vs. ^1^H-NMR (CDCl_3_, 293 K): δ 1.44–1.52 (m, 1H, CH=CHC*H*_2_), 1.59–1.79 (m, 6H, (C*H*_2_)_3_CH_2_CHCO), 2.05–2.11 (m, 1H, CH=CHC*H*_2_), 2.16–2.22 (m, 1H, C*H*_2_CHCO), 2.33–2.41 (m, 1H, C*H*_2_CHCO), 5.63–5.68 (m, 1H, CH_2_CH=C*H*), 5.72–5.77 (m, 1H, C*H*=CHCHCO), 5.91–5.95 (m, 1H, C*H*CO), 7.46 (t, 2H, *J* = 7.7 Hz, C_6_*H*_5_), 7.57 (t, 1H, *J* = 7.4 Hz, C_6_*H*_5_), 8.07–8.09 (m, 2H, C_6_*H*_5_). ^13^C-NMR (CDCl_3_, 293 K): δ 23.4, 25.9, 26.4, 28.8, 35.1, 73.0, 128.3, 129.6, 129.9, 130.7, 130.8, 132.7, 166.0. GC-MS (70 eV), *m*/*z*: 230 ([M^+^], 3), 202 (10), 149 (1), 123 (6), 105 (100), 93 (10), 77 (44), 67 (9), 51 (10), 41 (8). Elemental analysis (%) calculated for C_15_H_18_O_2_: C, 78.23; H, 7.88; found: C, 78.83; H, 7.81.

### 3.3. Crystallographic Data Collection and Refinement

Crystals suitable for the X-ray diffraction experiment were recrystallized from an acetone solution. A crystalline specimen of **1** was carefully separated from a conglomerate and turned out to be a very thin, clear, reddish-brown plate. The sample was gently picked up with a microloop wetted with paratone oil and placed on the top of the goniometer head of a kappa-geometry Oxford Diffraction Gemini EOS diffractometer, equipped with a 2 K × 2 K CCD area detector and sealed-tube Enhance (Mo) and (Cu) X-ray sources. Two different data collections were performed at room temperature by means of the *ω*-scan technique, using graphite-monochromated Cu and Mo *K*_α_ radiations (λ = 1.54184 and 0.71073 Å, respectively) in a 1024 × 1024 pixel mode and 2 × 2 pixel binning. Data collected under the Mo radiation [296.9 (9) K] afforded a better final solution and are those reported in the present work. The raw intensities were corrected for absorption, Lorentz, and polarization effects. With respect to absorption, an empirical multi-scan absorption correction based on equivalent reflections was applied by means of the scaling algorithm *SCALE3 ABSPACK*.

Final unit cell parameters were determined by least-squares refinement of 21,955 reflections picked during the whole experiment. Data collection, reduction, and finalization were performed with the CrysAlis Pro suite [94]. The structure was solved by intrinsic phasing in the *P* 2_1_/*c* space group with SHELXT [95], and refined by full-matrix least-squares methods based on *F*_o_^2^ with SHELXL [91] software integrated with the OLEX2 program [96]; there were no atoms sitting in special positions. During the refinement, we realized that almost all atoms [C(9) to C(14)] of the hexyloxy residue were disordered. Luckily, we were able to model the disorder by means of two alternative arrangements of the aliphatic chain, with SOFs constrained to sum to unity. The final occupancies turned out to be 0.705 and 0.295. The model of the aliphatic chain was further improved by imposing some additional restraints (DELU, SIMU, SADI, and RIGU) on the disordered atoms. In the end, the thermal factors of the terminal atoms in one arrangement were quite high, but no atoms needed to be split. The positions of all atoms (including the H atoms of the bis-pyrazolyl moiety) were identified by difference Fourier maps; non-hydrogen atoms were refined anisotropically. On the opposite side, H atoms of the disordered part of the molecule were added in calculated positions with *U*_iso_ values calculated from the *U*_eq_ of the pertinent carbon atoms. 

The crystallographic .cif file containing data for **1** was deposited at the Cambridge Crystallographic Data Center (CCDC no. 2110450). Data can be obtained free of charge via https://www.ccdc.cam.ac.uk/structures/ (accessed on 17 September 2021). 

## 4. Conclusions

A new dimeric copper(II) bromide complex was prepared in acetonitrile by a reaction of CuBr_2_ with the hexyl bis(pyrazol-1-yl)acetate ligand (L^OHex^), obtained by the esterification of the related bis(pyrazolyl)carboxylic acid. The X-ray crystal structure investigation revealed that, in the solid state, the compound exists as a dimer of formula [Cu(L^OHex^)Br(μ-Br)]_2_, with the Br(1) ions binding two symmetry-related units to each other, in which the copper ion shows a distorted square pyramidal arrangement. This new complex is among the few so far reported mono- or di-nuclear bis-pyrazolyl acetate copper complexes with uncoordinated acetate moieties that show µ-bridging bromide ions in the Cu coordination sphere. The complex was successfully investigated as a catalyst for the synthesis of oxygenate allylic compounds via the Kharasch–Sosnovsky reaction, avoiding the use of any external agents and superstoichiometric amounts of reagents, preventing the generation of excessive waste. Indeed, the use of the new catalyst allowed for the implementation of old synthetic procedures by decreasing the amount to 0.5 mol%, presumably due to the dimeric structure of the complex. Moreover, the ratio between the alkene and the oxidant species was decreased to 3 to 1, broadening the applicability spectrum of this procedure to more expensive and less available starting materials, as well. In fact, superstoichiometric amounts of olefin and long reaction times in the order of days represent the limitations of the implementation of this procedure in the synthesis of highly valuable species. Considering the interest in the allylic oxidation of olefins as an important step in the synthesis of natural compounds, drugs, and industrial products, future efforts will be focused on the applicability of these compounds to other catalytic pathways in order to ensure their catalytic activity and strength.

## Figures and Tables

**Table 1:**
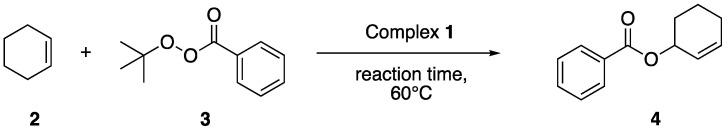
Catalytic activity of complex **1**.

Entry	1 mol%	2:3 Ratio	Reaction Time (h)	Yield (%) ^a^ 4
*1a*	5	5:1	6	73
*1b*	5	5:1	24	95
*1c*	5	3:1	24	79
*1d*	1	5:1	24	77
*1e*	1	3:1	24	74
*1f*	0.5	10:1	24	95
*1g*	0.5	5:1	24	90
*1h*	0.5	3:1	24	90
*1i*	0.5	1:1	48	33

^a^ Yield of pure isolated product.

**Table 2:** Catalytic activity data (catalysts, reaction conditions, and yield) obtained in Kharasch–Sosnovsky reactions using selected copper-based compounds as catalysts.

	Catalyst Type	Reaction Conditions	Yield (%)	Ref.
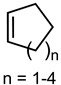	Cu(I) and Cu(II) salts	5 mol% cat., 32–48 h, PhNHNH_2_ (6 μL), CH_3_CN (3 mL), reflux	85 (*n* = 1) 92 (*n* = 2) 76 (*n* = 4)	[76]
	Cu(CH_3_CN)_4_PF_6_ immobilized on halloysite	20 mg cat., 7–45 h, CH_3_CN (3 mL), r.t.	67 (*n* = 1) 90 (*n* = 2) 80 (*n* = 4)	[77]
	Cu(I) and Cu(II) complexes	2.5 mol% Cu salt, 5 mol% chiral ligand, 50–81 h, PhNHNH_2_ (2 μL), HZSM-5 (5 mg), acetone, 0 °C	75 (*n* = 1) 85 (*n* = 3) 75 (*n* = 4)	[78]
	Cu(II) complexes	1 mol% Cu(OTf)_2_, 1.1 mol% chiral ligand, 0.5–168 h, 1.1 mol% PhNHNH_2_, acetone, r.t.	78 (*n* = 1) 82 (*n* = 2) 81 (*n* = 3) 0 (*n* = 4)	[79]
	CuBr	10 mol% CuBr, 18 h, dichlorobenzene, 70 °C, inert atm.	70 (*n* = 2)	[9]

**Table 3:** Bond lengths (Å) and angles (deg) for **1**.

**Bond**	**Bond Length (Å)**	**Bond**	**Bond Length (Å)**
Br(1)−Cu(1)	2.4302 (4)	Br(2)−Cu(1)	2.3687 (6)
Br(1)−Cu(1) ^I^	2.7600 (5)	Cu(1)−N(1)	2.017 (2)
Cu(1)−N(3)	2.042 (3)	N(1)−N(2)	1.357 (3)
N(3)−N(4)	1.369 (3)	N(1)−C(3)	1.330 (4)
N(2)−C(1)	1.342 (4)	N(3)−C(6)	1.305 (4)
N(4)−C(4)	1.338 (4)	N(2)−C(7)	1.445 (3)
N(4)−C(7)	1.434 (4)	C(1)−C(2)	1.344 (5)
C(2)−C(3)	1.384 (5)	C(4)−C(5)	1.355 (5)
C(5)−C(6)	1.399 (5)	C(7)−C(8)	1.533 (4)
O(1)−C(8)	1.198 (3)	O(2)−C(8)	1.310 (4)
**Bond**	**Bond Angle (°)**	**Bond**	**Bond Angle (°)**
Cu(1)−Br(1)−Cu(1) ^I^	93.544 (13)	Br(1)−Cu(1)−Br(1) ^I^	86.456 (13)
N(1)−Cu(1)−Br(1) ^I^	93.87 (7)	N(3)−Cu(1)−Br(1) ^I^	97.29 (7)
Br(2)−Cu(1)−Br(1)	93.260 (18)	N(1)−Cu(1)−Br(1)	176.35 (7)
N(3)−Cu(1)−Br(2)	157.34 (7)	N(1)−Cu(1)−N(3)	85.69 (10)
N(1)−Cu(1)−Br(2)	90.16 (8)	N(3)−Cu(1)−Br(1)	90.67 (7)
N(2)−N(1)−Cu(1)	122.68 (17)	N(4)−N(3)−Cu(1)	121.3 (2)
N(1)−N(2)−C(7)	118.8 (2)	N(3)−N(4)−C(7)	119.4 (2)
N(1)−C(3)−C(2)	110.6 (3)	N(3)−C(6)−C(5)	111.5 (3)
C(1)−N(2)−N(1)	111.2 (2)	C(4)−N(4)−N(3)	110.6 (3)
N(2)−C(1)−C(2)	107.3 (3)	N(4)−C(4)−C(5)	107.9 (3)
N(4)−C(7)−N(2)	110.1 (2)	C(1)−C(2)−C(3)	106.2 (3)
C(4)−C(5)−C(6)	104.8 (4)	O(2)−C(8)−C(7)	108.8 (2)
O(1)−C(8)−C(7)	124.6 (3)	O(1)−C(8)−O(2)	126.6 (3)

^I^ symmetry code: 1–x,1–y,1–z.

## Data Availability

The data presented in this study are available from the authors on request.

## Supplementary Materials

The following are available online: Table S1: Summary of crystal data and structure refinement for compound **1**; Table S2: Intermolecular contacts in **1**; Figure S1: ORTEP drawing of the asymmetric unit of **1**; Figure S2: ORTEP representation of the dimeric complex **1**, showing part of the selected numbering scheme; Figure S3: Packing diagrams for **1**; Figures S4–S18: ^1^H-NMR, ^13^C-NMR, FT-IR, and ESI-MS spectra.

## Sample Availability

Samples of the compounds **1**–**6** are available from the authors on request.

## References

[1] White M.C. (2012). C–H Bond Functionalization & Synthesis in the 21st Century: A Brief History and Prospectus. Synlett.

[2] Wencel-Delord J., Dröge T., Liu F., Glorius F. (2011). Towards mild metal-catalyzed C–H bond activation. Chem. Soc. Rev..

[3] Newhouse T., Baran P.S. (2011). If C–H Bonds Could Talk: Selective C-H Bond Oxidation. Angew. Chem. Int. Ed. Engl..

[4] Bergman R.G. (2007). C–H activation. Nature.

[5] Bregeault J.M. (2003). Transition-metal complexes for liquid-phase catalytic oxidation: Some aspects of industrial reactions and of emerging technologies. Dalton Trans..

[6] Ullmann F., Wolfgang G., Yamamoto Y.S., Campbell F.T., Pfefferkorn R., Rounsaville J. (1985). Ullmann’s Encyclopedia of Industrial Chemistry.

[7] Rawlinson D.J., Sosnovsky G. (1972). One-Step Substitutive Acyloxylation at Carbon. Part I. Reactions Involving Peroxides. Synthesis.

[8] García-Cabeza A.L., Moreno-Dorado F.J., Ortega M.J., Guerra F.M. (2016). Copper-Catalyzed Oxidation of Alkenes and Heterocycles. Synthesis.

[9] Zhu N., Qian B., Xiong H., Bao H. (2017). Copper-catalyzed regioselective allylic oxidation of olefins via C–H activation. Tetrahedron Lett..

[10] Andrus M.B., Lashley J.C. (2002). Copper catalyzed allylic oxidation with peresters. Tetrahedron.

[11] Kharasch M.S., Sosnovsky G. (1958). The Reactions of t-Butyl Perbenzoate and Olefins—A Stereospecific Reaction. J. Am. Chem. Soc..

[12] Kharasch M., Fono A. (1958). Communications-A New Method of Introducing Peroxy Groups into Organic Molecules. J. Org. Chem..

[13] Kharasch M., Sosnovsky G., Yang N. (1959). Reactions of t-butyl Peresters. I. The reaction of Peresters with olefins1. J. Am. Chem. Soc..

[14] Fraunhoffer K.J., Knochel P. (2014). 7.05 Oxidation Adjacent to CC Bonds. Comprehensive Organic Synthesis.

[15] Weidmann V., Maison W. (2013). Allylic Oxidations of Olefins to Enones. Synthesis.

[16] McLaughlin E.C., Choi H., Wang K., Chiou G., Doyle M.P. (2009). Allylic Oxidations Catalyzed by Dirhodium Caprolactamate via Aqueous tert-Butyl Hydroperoxide: The Role of the tert-Butylperoxy Radical. J. Org. Chem..

[17] Delcamp J.H., White M.C. (2006). Sequential Hydrocarbon Functionalization: Allylic C–H Oxidation/Vinylic C–H Arylation. J. Am. Chem. Soc..

[18] Chen M.S., White M.C. (2004). A Sulfoxide-Promoted, Catalytic Method for the Regioselective Synthesis of Allylic Acetates from Monosubstituted Olefins via C–H Oxidation. J. Am. Chem. Soc..

[19] Catino A.J., Forslund R.E., Doyle M.P. (2004). Dirhodium(II) Caprolactamate: An Exceptional Catalyst for Allylic Oxidation. J. Am. Chem. Soc..

[20] Eames J., Watkinson M. (2001). Catalytic allylic oxidation of alkenes using an asymmetric Kharasch-Sosnovsky reaction. Angew. Chem. Int. Ed. Engl..

[21] Brunel J.M., Legrand O., Buono G. (1999). Recent advances in asymmetric copper allylic oxidation of olefins. Comptes Rendus Acad. Sci. Ser. II C.

[22] Chemler S.R. (2015). Copper catalysis in organic synthesis. Beilstein J. Org. Chem..

[23] Nakamura A., Nakada M. (2013). Allylic Oxidations in Natural Product Synthesis. Synthesis.

[24] Tan Q., Hayashi M. (2009). Asymmetric Desymmetrization of 4,5-Epoxycyclohex-1-ene by Enantioselective Allylic Oxidation. Org. Lett..

[25] Tanaka T., Tan Q., Kawakubo H., Hayashi M. (2011). Formal Total Synthesis of (−)-Oseltamivir Phosphate. J. Org. Chem..

[26] Badreddine A., Karym E.M., Zarrouk A., Nury T., El Kharrassi Y., Nasser B., Cherkaoui Malki M., Lizard G., Samadi M. (2015). An expeditious synthesis of spinasterol and schottenol, two phytosterols present in argan oil and in cactus pear seed oil, and evaluation of their biological activities on cells of the central nervous system. Steroids.

[27] Rahman F.U., Rahman A.U., Tan T.W. (2010). Direct Cu-Catalyzed Allylic Acetoxylation of Δ5-Steroids at 7-Position. J. Chin. Chem. Soc..

[28] Zhang X., Jiang W., Sui Z. (2003). Concise Enantioselective Syntheses of Quinolactacins A and B through Alternative Winterfeldt Oxidation. J. Org. Chem..

[29] Patin A., Kanazawa A., Philouze C., Greene A.E., Muri E., Barreiro E., Costa P.C.C. (2003). Highly Stereocontrolled Synthesis of Natural Barbacenic Acid, Novel Bisnorditerpene from Barbacenia flava. J. Org. Chem..

[30] Ginotra S.K., Singh V.K. (2006). Enantioselective oxidation of olefins catalyzed by chiral copper bis(oxazolinyl)pyridine complexes: A reassessment. Tetrahedron.

[31] Aldea L., Garcia J.I., Mayoral J.A. (2012). Multiphase enantioselective Kharasch-Sosnovsky allylic oxidation based on neoteric solvents and copper complexes of ditopic ligands. Dalton Trans..

[32] Tan Q., Hayashi M. (2008). Novel N,N-bidentate ligands for enantioselective copper(I)-catalyzed allylic oxidation of cyclic olefins. Adv. Synth. Catal..

[33] Alvarez L.X., Christ M.L., Sorokin A.B. (2007). Selective oxidation of alkenes and alkynes catalyzed by copper complexes. Appl. Catal. A Gen..

[34] Walling C., Zavitsas A.A. (1963). The Copper-Catalyzed Reaction of Peresters with Hydrocarbons. J. Am. Chem. Soc..

[35] Kochi J.K., Mains H.E. (1965). Studies on the Mechanism of the Reaction of Peroxides and Alkenes with Copper Salts. J. Org. Chem..

[36] Beckwith A.L.J., Zavitsas A.A. (1986). Allylic Oxidations by Peroxy Esters Catalyzed by Copper Salts. The Potential for Stereoselective Syntheses. J. Am. Chem. Soc..

[37] Andrus M.B., Argade A.B., Chen X., Pamment M.G. (1995). The asymmetric kharasch reaction. Catalytic enantioselective allylic acyloxylation of olefins with chiral copper(I) complexes and tert-butyl perbenzoate. Tetrahedron Lett..

[38] Smith K., Hupp C.D., Allen K.L., Slough G.A. (2005). Catalytic allylic amination versus allylic oxidation: A mechanistic dichotomy. Organometallics.

[39] Mayoral J.A., Rodríguez-Rodríguez S., Salvatella L. (2008). Theoretical insights into enantioselective catalysis: The mechanism of the Kharasch-Sosnovsky reaction. Chem. Eur. J..

[40] Beck A., Weibert B., Burzlaff N. (2001). Monoanionic N,N,O-scorpionate ligands and their iron(II) and zinc(II) complexes: Models for mononuclear active sites of non-heme iron oxidases and zinc enzymes. Eur. J. Inorg. Chem..

[41] Otero A., Fernandez-Baeza J., Tejeda J., Antinolo A., Carrillo-Hermosilla F., Diez-Barra E., Lara-Sanchez A., Fernandez-Lopez M., Lanfranchi M., Pellinghelli M.A. (1999). Syntheses and crystal structures of lithium and niobium complexes containing a new type of monoanionic “scorpionate” ligand. J. Chem. Soc. Dalton Trans..

[42] Burzlaff N. (2008). Tripodal N,N,O-ligands for metalloenzyme models and organometallics. Advances in Inorganic Chemistry.

[43] Honrado M., Sobrino S., Fernández-Baeza J., Sánchez-Barba L.F., Garcés A., Lara-Sánchez A., Rodríguez A.M. (2019). Synthesis of an enantiopure scorpionate ligand by a nucleophilic addition to a ketenimine and a zinc initiator for the isoselective ROP of rac-lactide. Chem. Commun..

[44] Burzlaff N., Hegelmann I., Weibert B. (2001). Bis(pyrazol-1-yl)acetates as tripodal “scorpionate” ligands in transition metal carbonyl chemistry: Syntheses, structures and reactivity of manganese and rhenium carbonyl complexes of the type [LM(CO)3] (L = bpza, bdmpza). J. Organomet. Chem..

[45] Paul T., Rodehutskors P.M., Schmidt J., Burzlaff N. (2016). Oxygen Atom Transfer Catalysis with Homogenous and Polymer-Supported N,N- and N,N,O-Heteroscorpionate Dioxidomolybdenum(VI) Complexes. Eur. J. Inorg. Chem..

[46] Fischer N.V., Türkoglu G., Burzlaff N. (2009). Scorpionate complexes suitable for enzyme inhibitor studies. Curr. Bioact. Compd..

[47] Costas M., Mehn M.P., Jensen M.P., Que L. (2004). Dioxygen Activation at Mononuclear Nonheme Iron Active Sites: Enzymes, Models, and Intermediates. Chem. Rev..

[48] Parkin G. (2004). Synthetic Analogues Relevant to the Structure and Function of Zinc Enzymes. Chem. Rev..

[49] Pellei M., Bagnarelli L., Luciani L., Del Bello F., Giorgioni G., Piergentili A., Quaglia W., De Franco M., Gandin V., Marzano C. (2020). Synthesis and Cytotoxic Activity Evaluation of New Cu(I) Complexes of Bis(pyrazol-1-yl) Acetate Ligands Functionalized with an NMDA Receptor Antagonist. Int. J. Mol. Sci..

[50] Giorgetti M., Tonelli S., Zanelli A., Aquilanti G., Pellei M., Santini C. (2012). Synchrotron radiation X-ray absorption spectroscopic studies in solution and electrochemistry of a nitroimidazole conjugated heteroscorpionate copper(II) complex. Polyhedron.

[51] Pellei M., Papini G., Trasatti A., Giorgetti M., Tonelli D., Minicucci M., Marzano C., Gandin V., Aquilanti G., Dolmella A. (2011). Nitroimidazole and glucosamine conjugated heteroscorpionate ligands and related copper(II) complexes. Syntheses, biological activity and XAS studies. Dalton Trans..

[52] Morelli M.B., Amantini C., Santoni G., Pellei M., Santini C., Cimarelli C., Marcantoni E., Petrini M., Del Bello F., Giorgioni G. (2018). Novel antitumor copper(ii) complexes designed to act through synergistic mechanisms of action, due to the presence of an NMDA receptor ligand and copper in the same chemical entity. New J. Chem..

[53] Pellei M., Gandin V., Cimarelli C., Quaglia W., Mosca N., Bagnarelli L., Marzano C., Santini C. (2018). Syntheses and biological studies of nitroimidazole conjugated heteroscorpionate ligands and related Cu(I) and Cu(II) complexes. J. Inorg. Biochem..

[54] Reiss A., Cioateră N., Dobrițescu A., Rotaru M., Carabet A.C., Parisi F., Gănescu A., Dăbuleanu I., Spînu C.I., Rotaru P. (2021). Bioactive Co(II), Ni(II), and Cu(II) Complexes Containing a Tridentate Sulfathiazole-Based (ONN) Schiff Base. Molecules.

[55] Otero A., Fernández-Baeza J., Lara-Sánchez A., Sánchez-Barba L.F. (2013). Metal complexes with heteroscorpionate ligands based on the bis(pyrazol-1-yl)methane moiety: Catalytic chemistry. Coord. Chem. Rev..

[56] Godau T., Bleifuß S.M., Müller A.L., Roth T., Hoffmann S., Heinemann F.W., Burzlaff N. (2011). Cu(i) catalysed cyclopropanation with enantiopure scorpionate type ligands derived from (+)-camphor or (−)-menthone. Dalton Trans..

[57] Jun Z., Braunstein P., Hor T.S.A. (2008). Highly selective chromium(III) ethylene trimerization catalysts with [NON] and [NSN] heteroscorpionate ligands. Organometallics.

[58] Zhang J., Li A., Hor T.S.A. (2009). Crystallographic revelation of the role of AIMe3 (in MAO) in Cr [NNN] pyrazolyl catalyzed ethylene trimerization. Organometallics.

[59] Otero A., Fernandez B., Antinolo A., Carrillo H., Tejeda J., Diez B., Lara S., Sanchez B., Lopez S., Ribeiro M.R. (2001). Polymerization of ethylene by the electrophilic heteroscorpionate-containing complexes [TiCl3(bdmpza)] and [TiCl2(bdmpza) {O(CH2)4Cl}] (bdmpza = bis(3,5-dimethylpyrazol-1-yl)acetate). Organometallics.

[60] Türkoglu G., Tampier S., Strinitz F., Heinemann F.W., Hübner E., Burzlaff N. (2012). Ruthenium Carbonyl Complexes Bearing Bis(pyrazol-1-yl)carboxylato Ligands. Organometallics.

[61] Rhinehart J.L., Manbeck K.A., Buzak S.K., Lippa G.M., Brennessel W.W., Goldberg K.I., Jones W.D. (2012). Catalytic Arene H/D Exchange with Novel Rhodium and Iridium Complexes. Organometallics.

[62] Kopf H., Holzberger B., Pietraszuk C., Huebner E., Burzlaff N. (2008). Neutral Ruthenium Carbene Complexes bearing N,N,O Heteroscorpionate Ligands: Syntheses and Activity in Metathesis Reactions. Organometallics.

[63] Datta A., Das K., Beyene B.B., Garribba E., Gajewska M.J., Hung C.-H. (2017). EPR interpretation and electrocatalytic H2 evolution study of bis(3,5-di-methylpyrazol-1-yl)acetate anchored Cu(II) and Mn(II) complexes. Mol. Catal..

[64] Tada M., Iwasawa Y. (2006). Advanced chemical design with supported metal complexes for selective catalysis. Chem. Commun..

[65] Dioos B.M.L., Vankelecom I.F.J., Jacobs P.A. (2006). Aspects of immobilisation of catalysts on polymeric supports. Adv. Synth. Catal..

[66] Burguete M.I., Fraile J.M., García J.I., García-Verdugo E., Herrerías C.I., Luis S.V., Mayoral J.A. (2001). Bis(oxazoline)copper complexes covalently bonded to insoluble support as catalysts in cyclopropanation reactions. J. Org. Chem..

[67] Padnya P., Shibaeva K., Arsenyev M., Baryshnikova S., Terenteva O., Shiabiev I., Khannanov A., Boldyrev A., Gerasimov A., Grishaev D. (2021). Catechol-Containing Schiff Bases on Thiacalixarene: Synthesis, Copper (II) Recognition, and Formation of Organic-Inorganic Copper-Based Materials. Molecules.

[68] Hübner E., Haas T., Burzlaff N. (2006). Synthesis and transition metal complexes of novel N,N,O scorpionate ligands suitable for solid phase immobilisation. Eur. J. Inorg. Chem..

[69] Hübner E., Türkoglu G., Wolf M., Zenneck U., Burzlaff N. (2008). Novel N,N,O scorpionate ligands and transition metal complexes thereof suitable for polymerisation. Eur. J. Inorg. Chem..

[70] Türkoglu G., Pubill Ulldemolins C., Müller R., Hübner E., Heinemann F.W., Wolf M., Burzlaff N. (2010). Bis(3,5-dimethyl-4-vinylpyrazol-1-yl)acetic Acid: A New Heteroscorpionate Building Block for Copolymers that Mimic the 2-His-1-carboxylate Facial Triad. Eur. J. Inorg. Chem..

[71] Mal D.D., Kundu J., Pradhan D. (2021). CuO{001} as the Most Active Exposed Facet for Allylic Oxidation of Cyclohexene via a Greener Route. ChemCatChem.

[72] Tetour D., Novotná M., Hodačová J. (2021). Enantioselective Henry Reaction Catalyzed by Copper(II) Complex of Bis(trans-cyclohexane-1,2-diamine)-Based Ligand. Catalysts.

[73] Sekar G., Dattagupta A., Singh V.K. (1998). Asymmetric Kharasch Reaction: Catalytic Enantioselective Allylic Oxidation of Olefins Using Chiral Pyridine Bis(diphenyloxazoline)—Copper Complexes and tert-Butyl Perbenzoate. J. Org. Chem..

[74] Gabrielli S., Pellei M., Venditti I., Fratoddi I., Battocchio C., Iucci G., Schiesaro I., Meneghini C., Palmieri A., Marcantoni E. (2020). Development of new and efficient copper(II) complexes of hexyl bis(pyrazolyl)acetate ligands as catalysts for allylic oxidation. Dalton Trans..

[75] ECHA European Chemicals Agency Phenylhydrazine. https://echa.europa.eu/it/registration-dossier/-/registered-dossier/14149/7/3/1.

[76] Samadi S., Ashouri A., Majidian S., Rashid H. (2020). Synthesis of new alkenyl iodobenzoate derivatives via Kharasch-Sosnovsky reaction using tert-butyl iodo benzoperoxoate and copper (I) iodide. J. Chem. Sci..

[77] Sadjadi S., Samadi S., Samadi M. (2019). Cu(CH3CN)4PF6 immobilized on halloysite as efficient heterogeneous catalyst for oxidation of allylic C–H bonds in olefins under mild reaction condition. Res. Chem. Intermed..

[78] Samadi S., Jadidi K., Samadi M., Ashouri A., Notash B. (2019). Designing chiral amido-oxazolines as new chelating ligands devoted to direct Cu-catalyzed oxidation of allylic C H bonds in cyclic olefins. Tetrahedron.

[79] Chelucci G., Loriga G., Murineddu G., Pinna G.A. (2002). Synthesis and application in asymmetric copper(I)-catalyzed allylic oxidation of a new chiral 1,10-phenanthroline derived from pinene. Tetrahedron Lett..

[80] Johnson C.K. (1976). ORTEP, Report ORNL–5138.

[81] Kozlevčar B., Pregelj T., Pevec A., Kitanovski N., Costa J.S., Van Albada G., Gamez P., Reedijk J. (2008). Copper complexes with the ligand methyl bis(3,5-dimethylpyrazol-1-yl)- acetate (Mebdmpza), generated by in situ methanolic esterification of bis(3,5-dimethylpyrazol-1-yl)acetic acid. Eur. J. Inorg. Chem..

[82] Quillian B., Lynch W.E., Padgett C.W., Lorbecki A., Petrillo A., Tran M. (2019). Syntheses and Crystal Structures of Copper(II) Bis(pyrazolyl)acetic Acid Complexes. J. Chem. Crystallogr..

[83] Romero M.A., Salas J.M., Quiros M., Sanchez M.P., Romero J., Martin D. (1994). Structural and magnetic studies on a bromine-bridged copper(II) dimer with 5,7-dimethyl[1,2,4]triazolo[1,5-a]pyrimidine. Inorg. Chem..

[84] Lavrenova L.G., Ikorskij V.N., Sheludyakova L.A., Naumov D.Y., Boguslavskij E.G. (2004). Co(II), Ni(II) and Cu(II) complexes with 1-(4-hydroxyphenyl)-1H-1,2,4-triazole. Russ. J. Coord. Chem..

[85] Dobrzanska L., Kleinhans D.J., Barbour L.J. (2008). Influence of the metal-to-ligand ratio on the formation of metal organic complexes. New J. Chem..

[86] Hoffmann A., Herres-Pawlis S. (2013). Dissection of Different Donor Abilities Within Bis(pyrazolyl)pyridinylmethane Transition Metal Complexes. Z. Anorg. Allg. Chem..

[87] Andreeva T.N., Lyakhov A.S., Ivashkevich L.S., Voitekhovich S.V., Grigoriev Y.V., Ivashkevich O.A. (2015). 1-(Furan-2-ylmethyl)-1H-tetrazole and its Copper(II) Complexes. Z. Anorg. Allg. Chem..

[88] Dyukova I.I., Kuz’menko T.A., Komarov V.Y., Sukhikh T.S., Vorontsova E.V., Lavrenova L.G. (2018). Coordination Compounds of Cobalt(II), Nickel(II), and Copper(II) Halides with 2-Methyl-1,2,4-Triazolo[1,5-a]benzimidazole. Russ. J. Coord. Chem..

[89] Allen F.H. (2002). The Cambridge Structural Database: A quarter of a million crystal structures and rising. Acta Crystallogr. Sect. B Struct. Sci..

[90] Macrae C.F., Bruno I.J., Chisholm J.A., Edgington P.R., McCabe P., Pidcock E., Rodriguez-Monge L., Taylor R., van de Streek J., Wood P.A. (2008). Mercury CSD 2.0—New features for the visualization and investigation of crystal structures. J. Appl. Crystallogr..

[91] Sheldrick G.M. (2015). Crystal structure refinement with SHELXL. Acta Crystallogr. Sect. C Struct. Chem..

[92] Addison A.W., Rao T.N., Reedijk J., van Rijn J., Verschoor G.C. (1984). Synthesis, structure, and spectroscopic properties of copper(II) compounds containing nitrogen–sulphur donor ligands; the crystal and molecular structure of aqua[1,7-bis(N-methylbenzimidazol-2′-yl)-2,6-dithiaheptane]copper(II) perchlorate. J. Chem. Soc. Dalton Trans..

[93] Konno T., Tokuda K., Sakurai J., Okamoto K.-I. (2000). Five-Coordinate Geometry of Cadmium(II) with Octahedral Bidentate-S,S Complex-Ligand cis(S)-[Co(aet)2(en)]+ (aet = 2-aminoethanethiolate): Synthesis, Crystal Structures and Interconversion of S-Bridged CoIIICdII Polynuclear Complexes. Bull. Chem. Soc. Jpn..

[94] (2021). CrysAlisPro 1.171.41.103a Rigaku Oxford Diffraction. https://www.rigaku.com/products/crystallography/crysalis.

[95] Sheldrick G.M. (2015). SHELXT—Integrated space-group and crystal-structure determination. Acta Crystallogr. Sect. A Found. Crystallogr..

[96] Dolomanov O.V., Bourhis L.J., Gildea R.J., Howard J.A.K., Puschmann H. (2009). OLEX2: A complete structure solution, refinement and analysis program. J. Appl. Crystallogr..

