# Supplementary material for: A New Dimeric Copper(II) Complex of Hexyl Bis(pyrazolyl)acetate Ligand as an Efficient Catalyst for Allylic Oxidations
Source: Molecules. 2021 Oct 16;26(20):6271. doi: 10.3390/molecules26206271 (PMC8541649; doi:10.3390/molecules26206271)
Supplement: Supplementary file 1 [file molecules-26-06271-s001.zip › molecules-1406092-supplementary.pdf]

molecules

Supporting Information

A new dimeric copper(II) complex of hexyl bis(pyrazolyl)acetate ligand as efficient catalyst for allylic oxidations

Luca Bagnarelli [1], Alessandro Dolmella [2], Carlo Santini [1], Riccardo Vallesi [1], Roberto Giacomantonio [1], Serena Gabrielli [1,*] and Maura Pellei [1,*]

1 School of Science and Technology, Chemistry Division, University of Camerino, via S. Agostino 1, 62032 Camerino, Italy; luca.bagnarelli@unicam.it (L.B.); carlo.santini@unicam.it (C.S.); riccardo.vallesi@unicam.it (R.V.); robert.giacomantonio@unicam.it (R.G.)
2 Department of Pharmaceutical and Pharmacological Sciences, University of Padova, via Marzolo 5, 35131 Padova, Italy; alessandro.dolmella@unipd.it
* Correspondence: serena.gabrielli@unicam.it (S.G.); maura.pellei@unicam.it (M.P.); Tel.: +39-0737-402219 (S.G.); +39-0737-402213 (M.P.)

Contents

Table S1: Summary of crystal data and structure refinement for compound **1**.
Table S2. Intermolecular contacts in **1**.
Figure S1: ORTEP drawing of the asymmetric unit of **1**, showing part of the selected numbering scheme.
Figure S2: ORTEP representation of the dimeric complex **1** showing part of the selected numbering scheme.
Figure S3. Packing diagrams for **1**.
Figure S4: ^{1}H-NMR of $HC(COOHex)(pz)_2$ (L^{OHex}) in $CDCl_3$.
Figure S5: ^{13}C-NMR of $HC(COOHex)(pz)_2$ (L^{OHex}) in $CDCl_3$.
Figure S6: FT-IR spectrum of $HC(COOHex)(pz)_2$ (L^{OHex}).
Figure S7: ESI-MS(+) spectrum of $HC(COOHex)(pz)_2$ (L^{OHex}).
Figure S8: FT-IR spectrum of $[Cu(L^{OHex})Br(\mu\text{-}Br)]_2$ (**1**).
Figure S9: ESI-MS(+) spectrum of $[Cu(L^{OHex})Br(\mu\text{-}Br)]_2$ (**1**).
Figure S10: ^{1}H-NMR of compound **4** in $CDCl_3$.
Figure S11: ^{13}C-NMR of compound **4** in $CDCl_3$.
Figure S12: FT-IR spectrum of compound **4**.
Figure S13: ^{1}H-NMR of compound **5** in $CDCl_3$.
Figure S14: ^{13}C-NMR of compound **5** in $CDCl_3$.
Figure S15: FT-IR spectrum of compound **5**.
Figure S16: ^{1}H-NMR of compound **6** in $CDCl_3$.
Figure S17: ^{13}C-NMR of compound **6** in $CDCl_3$.
Figure S18: FT-IR spectrum of compound **6**.

Table S1. Summary of crystal data and structure refinement for compound **1**.

Empirical formula	$C_{14}H_{20}N_4O_2CuBr_2$
Formula weight	499.70
Temperature / K	296.9(9)
Crystal system	monoclinic
Space group	$P2_1/c$
a / Å	13.0171(4)
b / Å	9.8111(2)
c / Å	15.8632(4)
α / °	90
β / °	110.248(3)
γ / °	90
Volume / Å^3	1900.73(9)
Z	4
ρ_{calc} g/cm^3	1.746
μ / mm^{-1}	5.366
F(000)	988.0
Crystal size / mm^3	0.6 × 0.4 × 0.01
Radiation	Mo Kα (λ = 0.71073)
Index ranges	−15 ≤ h ≤ 17, −13 ≤ k ≤ 12, −19 ≤ l ≤ 20
Reflections collected	22513
Independent reflections / R_{int}	4228 / 0.0437
Data / restraints / parameters	4228 / 78 / 293
Goodness-of-fit on F^2	1.058
Final R indexes [I>=2σ (I)]	R_1 = 0.0351, wR_2 = 0.0666
Largest diff. peak / hole / e Å^{-3}	0.50 / −0.46

[a] Goodness-of-fit = $[\Sigma w (F_o^2 - F_c^2)^2 / (N_{obsevns} - N_{params})]^{1/2}$, based on all data;. [b] $R_1 = \Sigma ||F_o| - |F_c|| / \Sigma |F_o|$;. [c] $wR_2 = [\Sigma w (F_o^2 - F_c^2)^2 / \Sigma w (F_o^2)^2]^{1/2}$.

Table S2. Intermolecular contacts in **1**.

Atom (A)	Atom (B)	A····B (Å)	Symmetry [a]
C(8)	Br(1)	3.37	1 – x , –1/2 + y, 3/2 – z
C(7)	Br(1)	3.43	1 – x , –1/2 + y, 3/2 – z
H(7)	Br(1)	2.91	1 – x , –1/2 + y, 3/2 – z
H(1)	Br(1)	2.93	x, –1 + y, z

[a] Symmetry operator of B atom; atom A belongs to asymmetric unit.

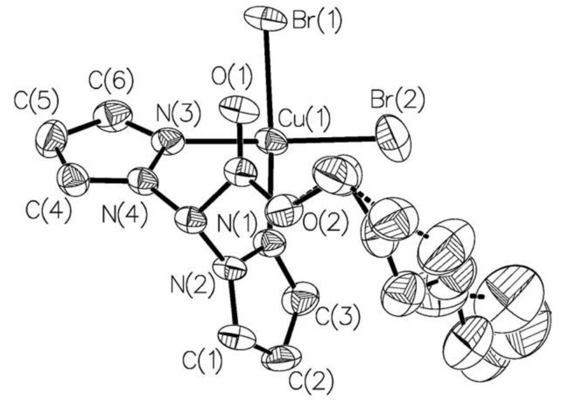

Figure S1. ORTEP drawing of the asymmetric unit of **1**, showing part of the selected numbering scheme. Hydrogen atoms omitted for clarity. The alternate arrangement of the disordered hexyloxy chain has dashed bonds. Thermal ellipsoids drawn at the 50% probability level.

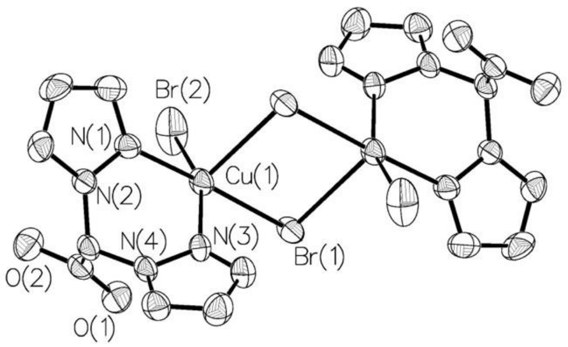

Figure S2. An ORTEP representation of the dimeric complex **1** showing part of the selected numbering scheme. Hydrogen atoms and disordered hexyloxy chains removed for clarity. Thermal ellipsoids drawn at the 50% probability level.

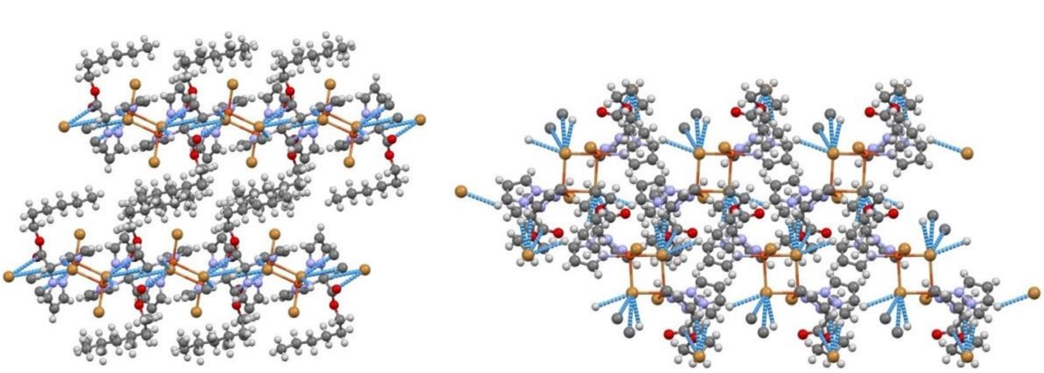

Figure S3. Packing diagrams for **1**. Left: the bi–dimensional network developing in the *bc* plane (view down the *a* axis). Right: the 'sandwich' motif created by the planes incorporating the hexyl aliphatic chains and the part of the molecules containing the Cu_2Br_2 tetramers developing along the *a* axis. Contacts colored in cyan.

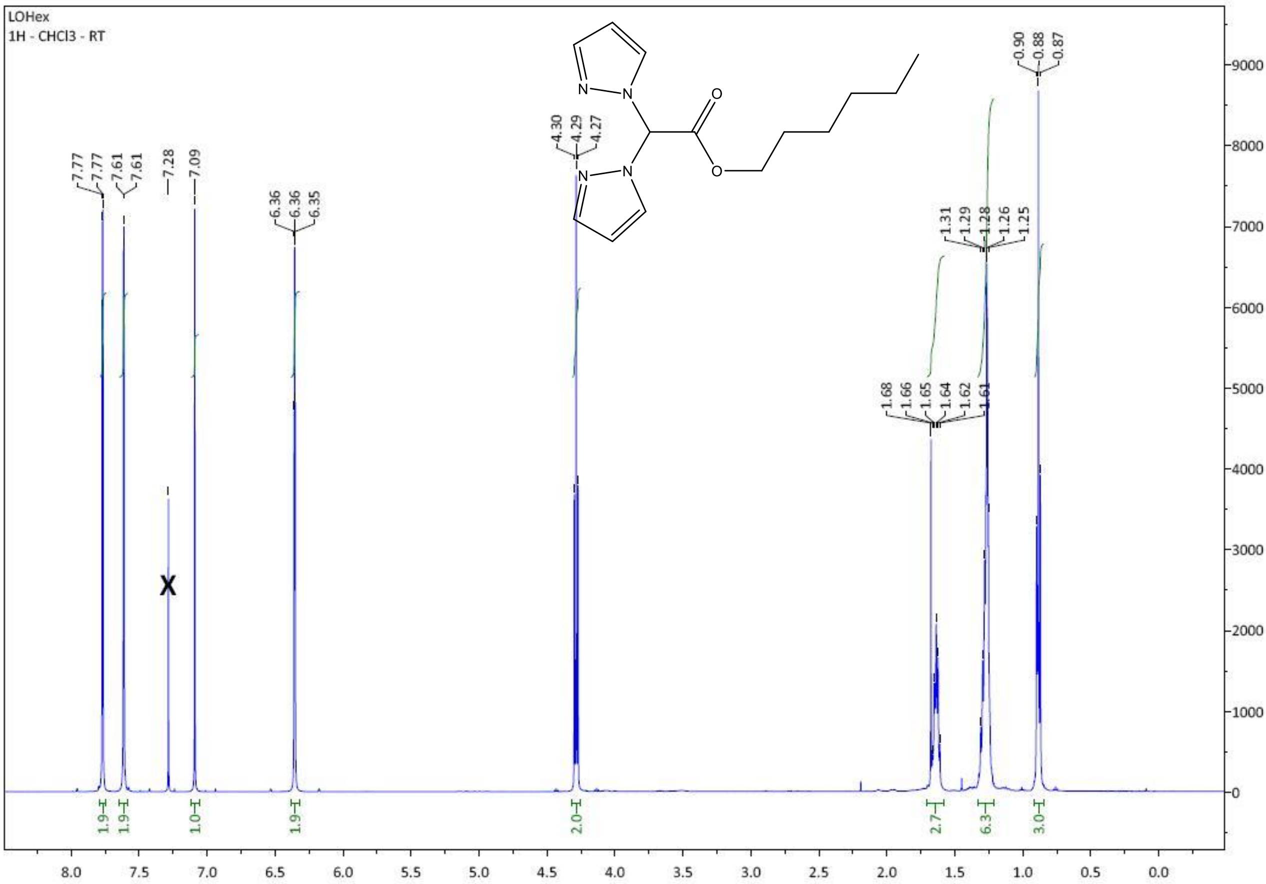

Figure S4. ^{1}H-NMR of HC(COOHex)(pz)$_2$ (L^{OHex}) in CDCl$_3$.

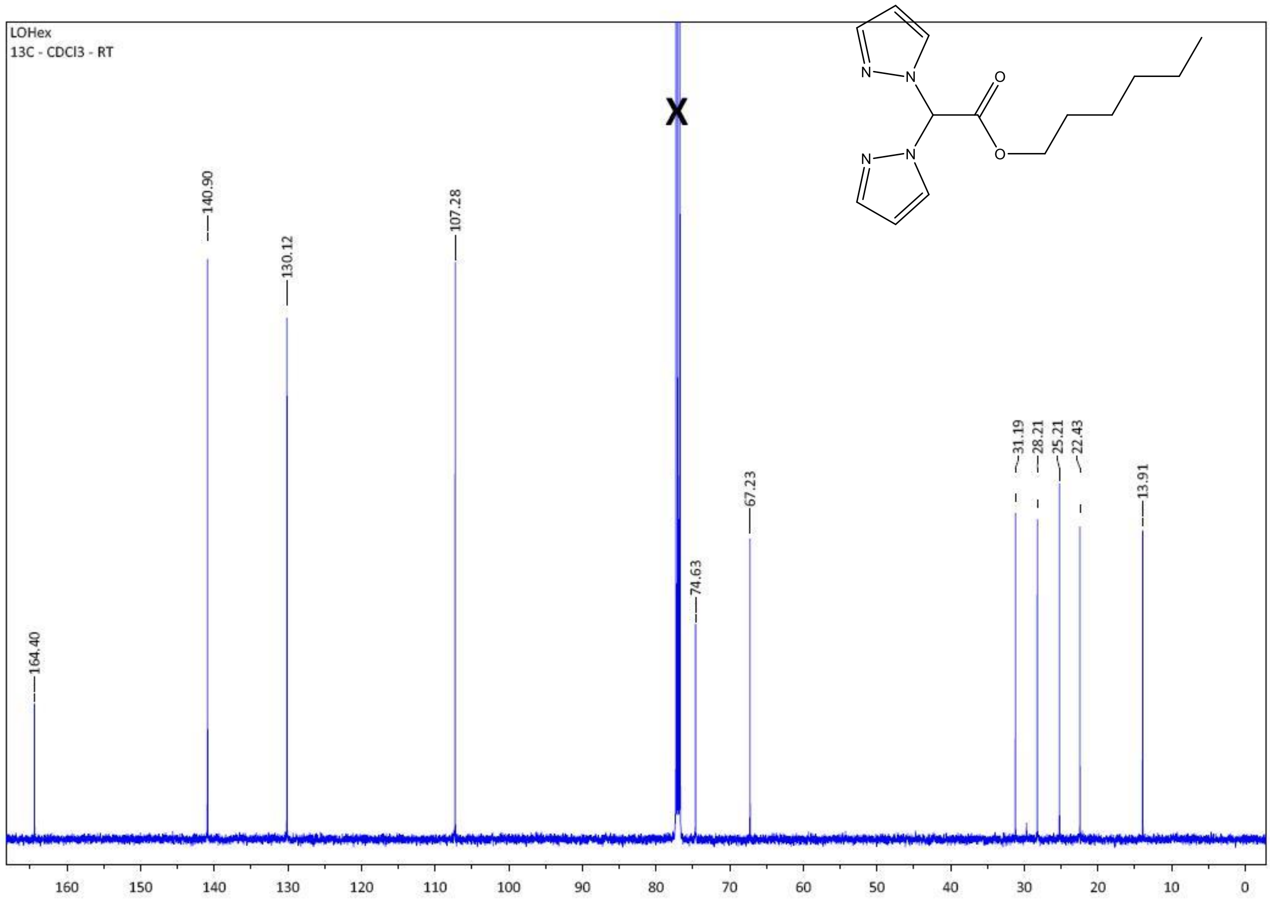

Figure S5. ^{13}C-NMR of HC(COOHex)(pz)$_2$ (L^{OHex}) in $CDCl_3$.

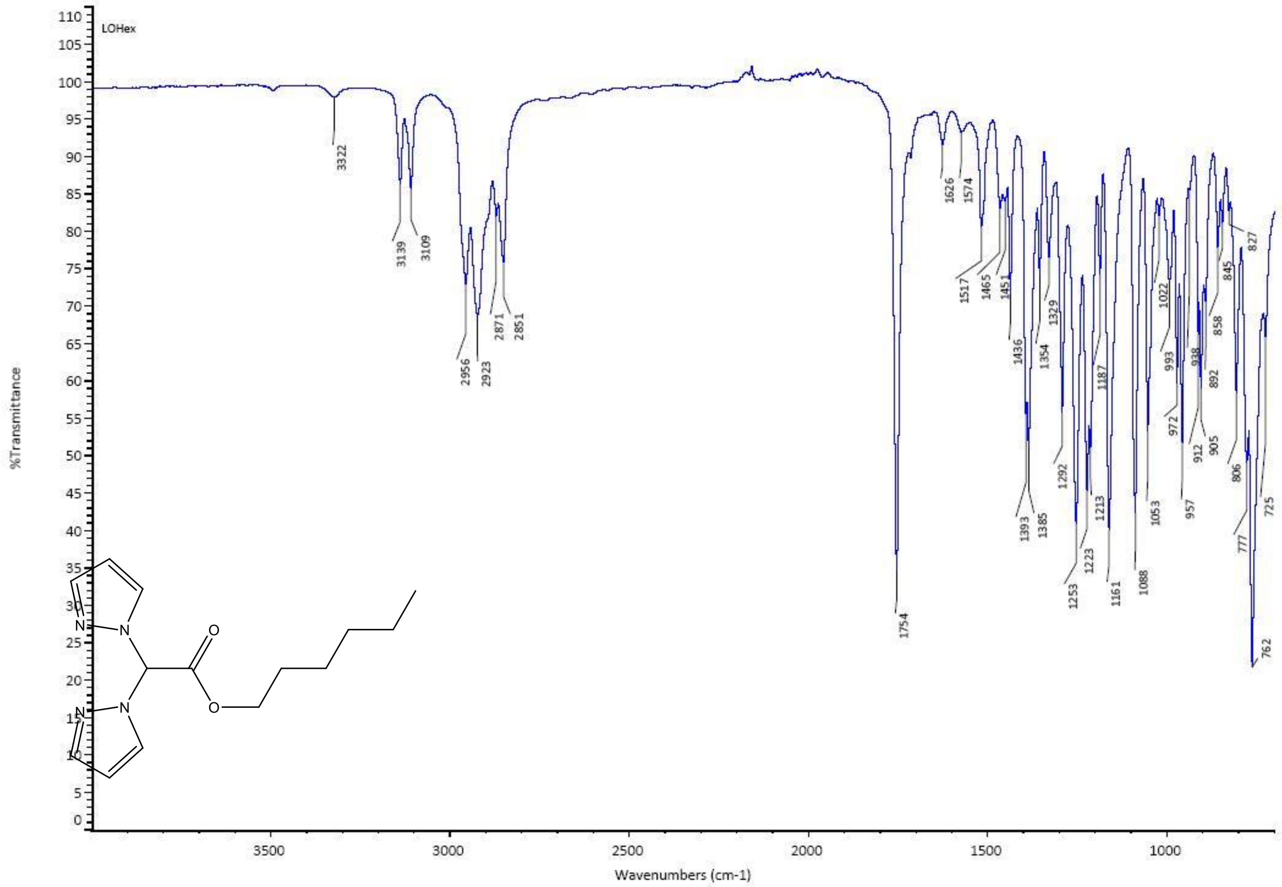

Figure S6. FT-IR spectrum of HC(COOHex)(pz)$_2$ (L^{OHex}).

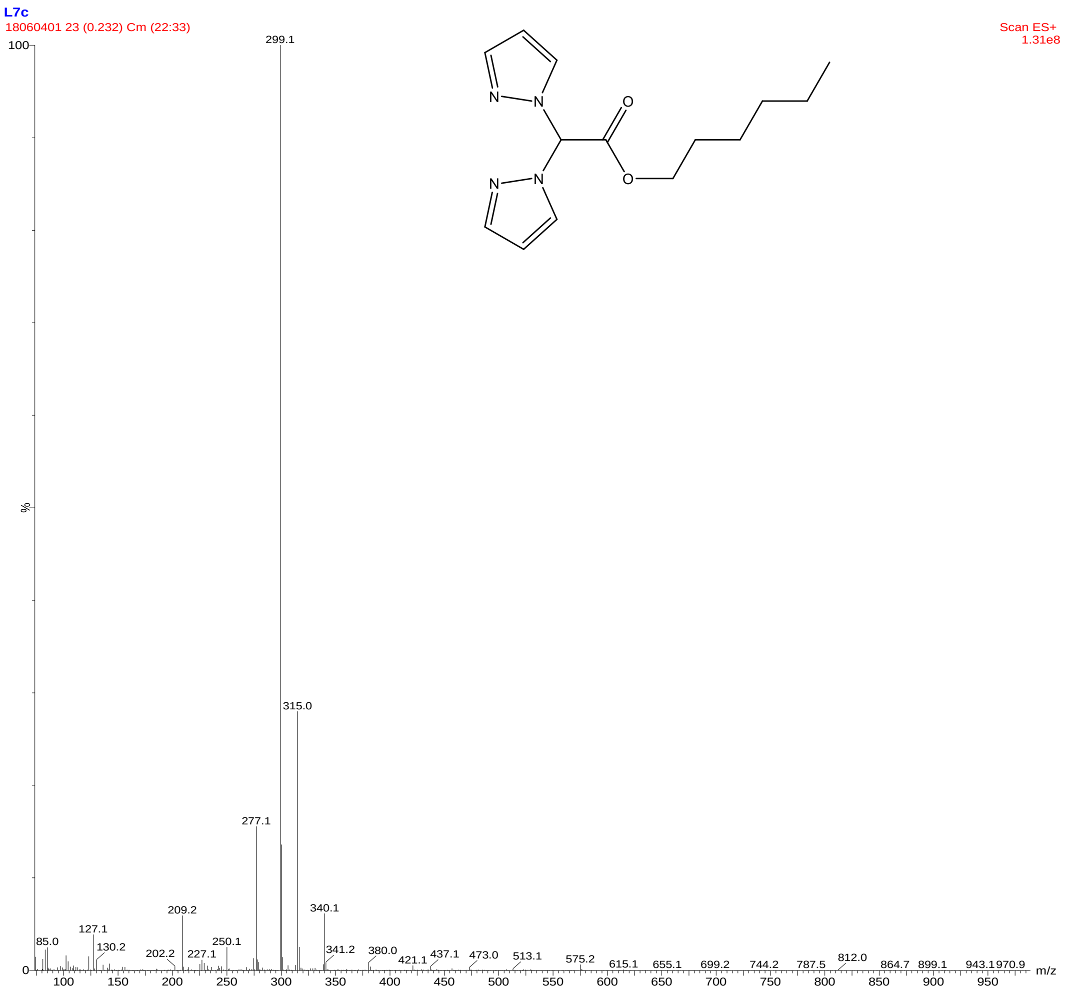

Figure S7. ESI-MS(+) spectrum of HC(COOHex)(pz)2 (L^{OHex}).

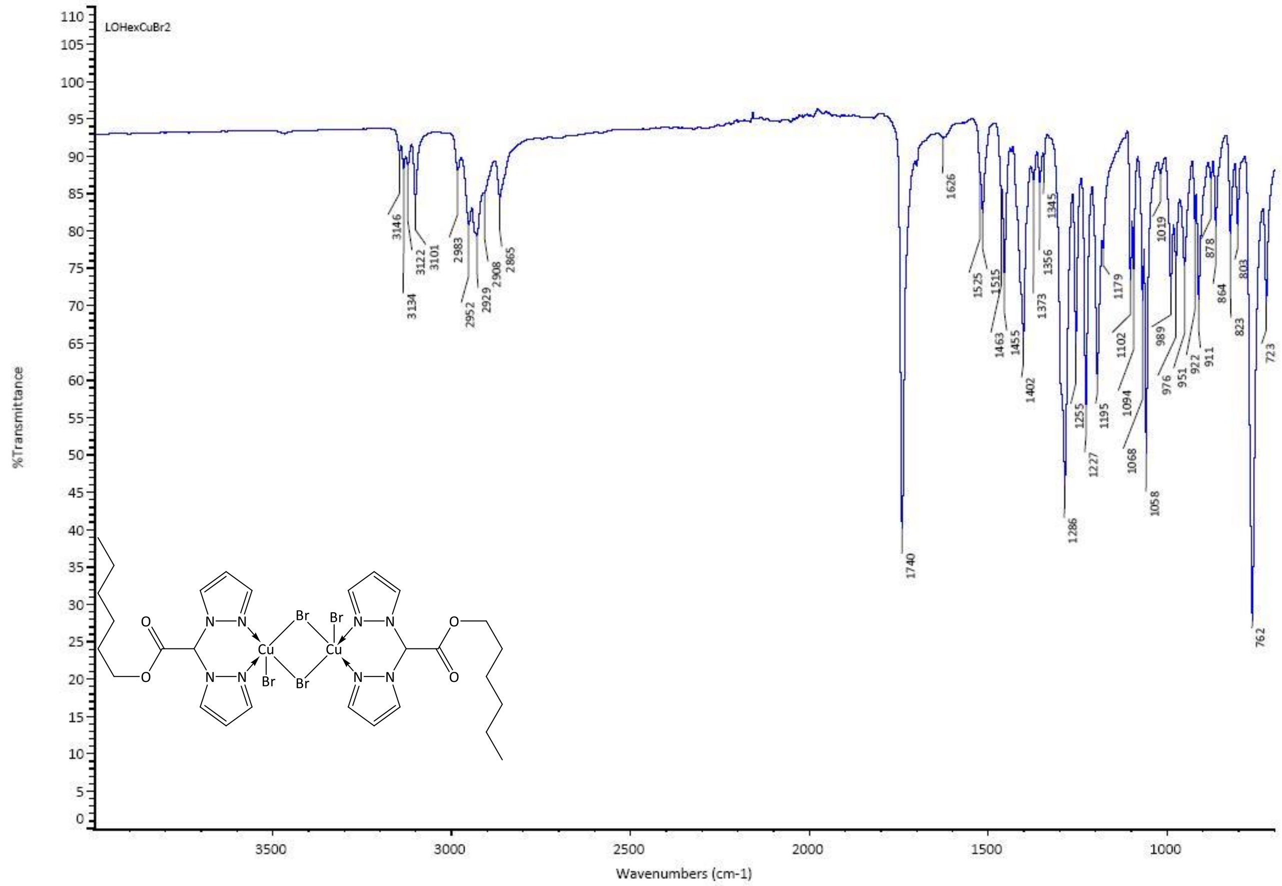

Figure S8. FT-IR spectrum of $[Cu(L^{OHex})Br(\mu\text{-}Br)]_2$ (**1**).

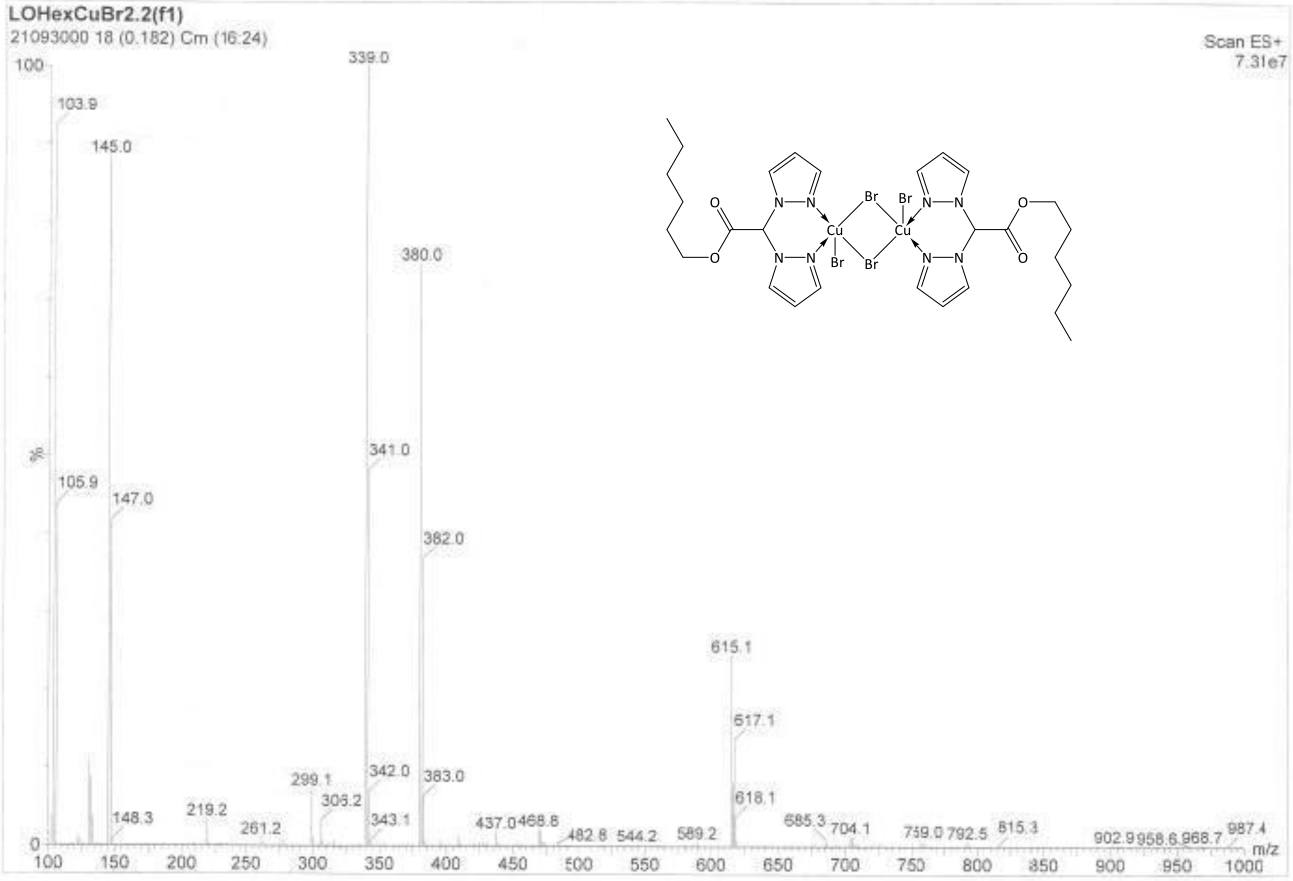

Figure S9. ESI-MS(+) spectrum of [Cu(L^{OHex})Br(μ-Br)]$_2$ (**1**).

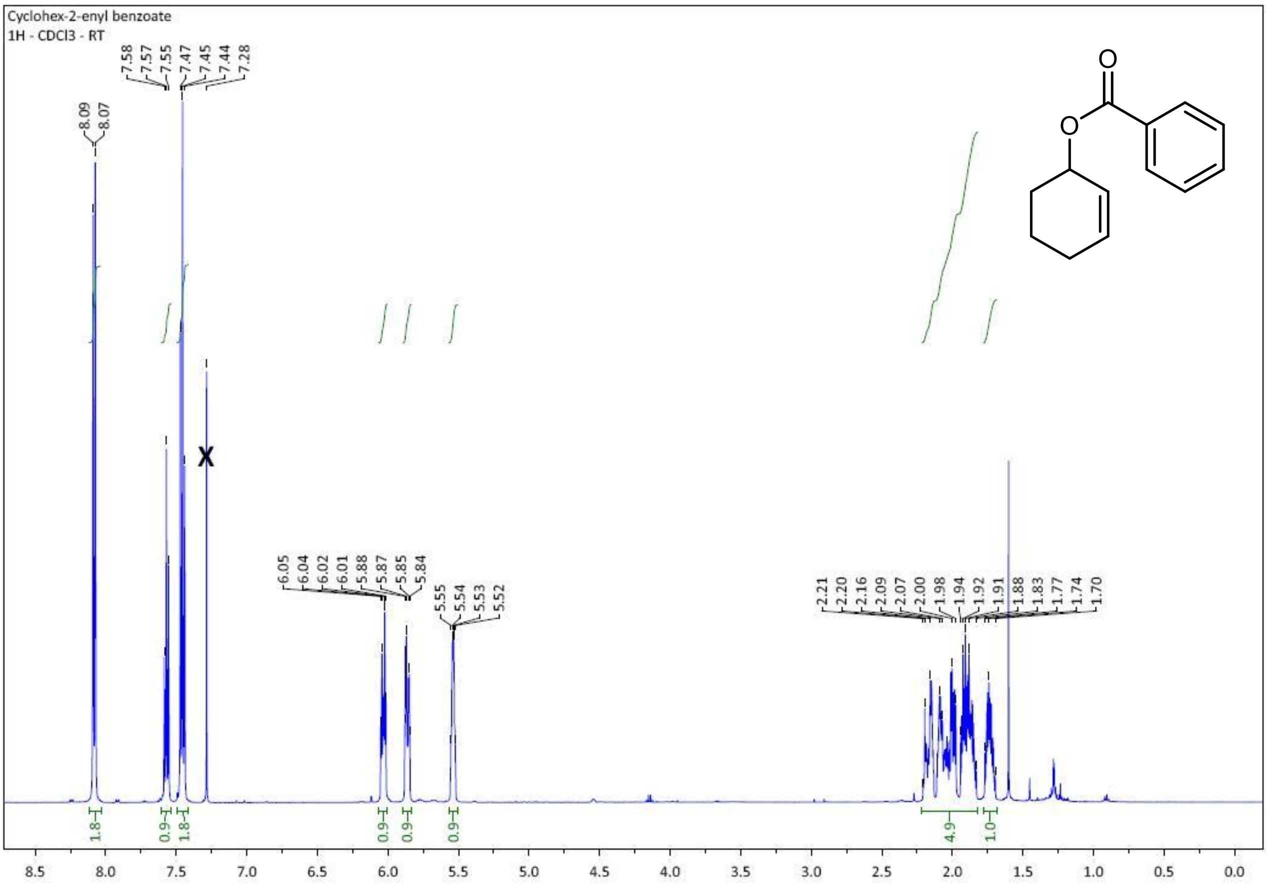

Figure S10. ^{1}H-NMR of compound **4** in $CDCl_3$.

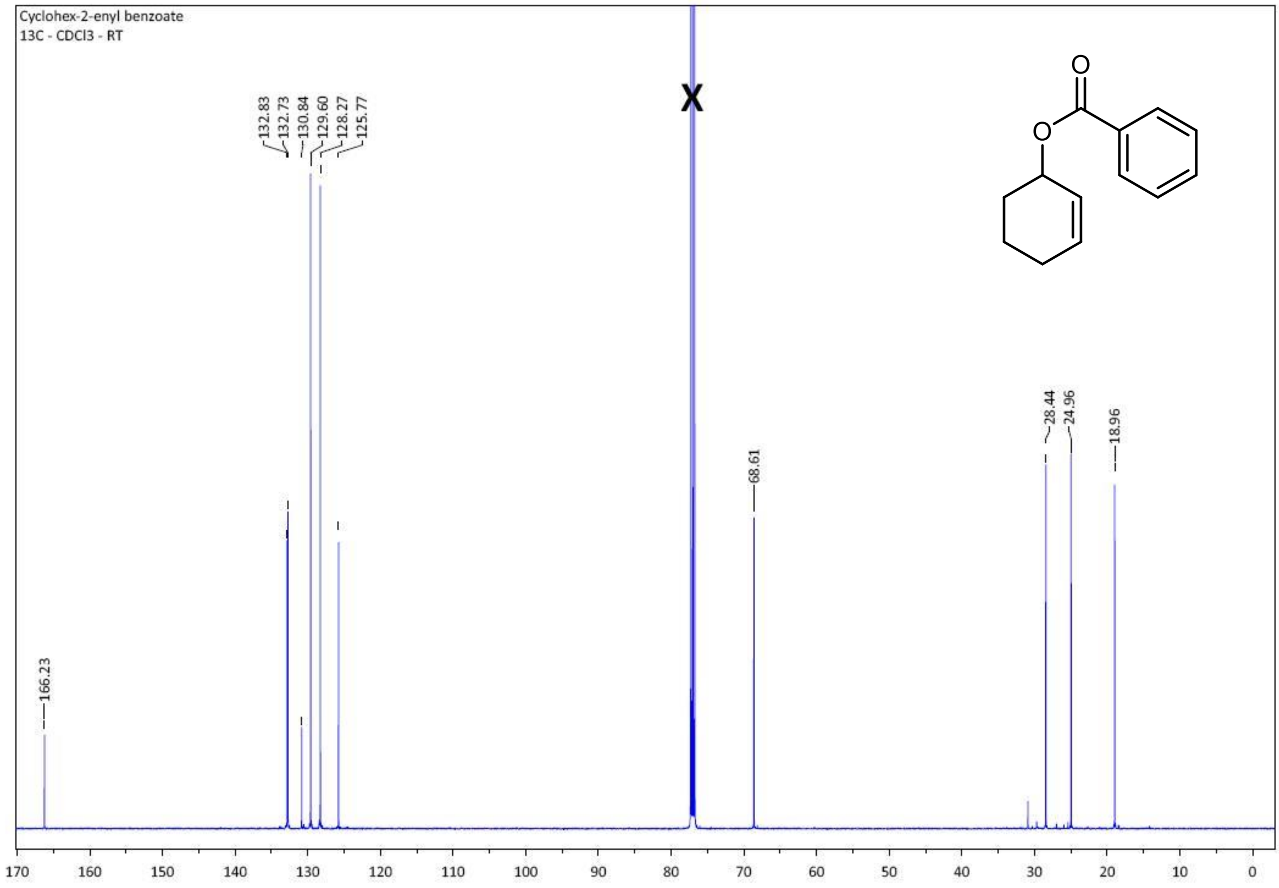

Figure S11. ^{13}C-NMR of compound **4** in $CDCl_3$.

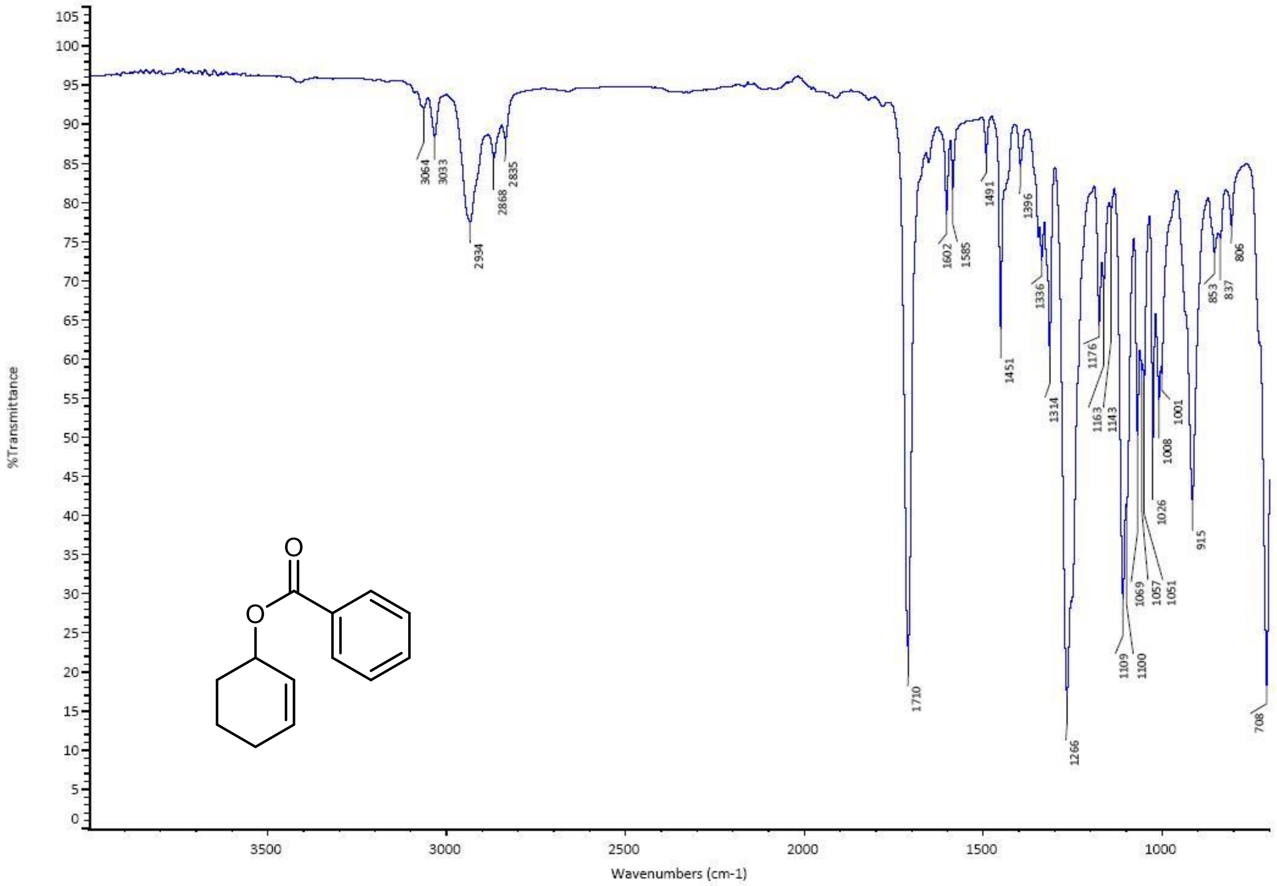

Figure S12. FT-IR spectrum of compound **4**.

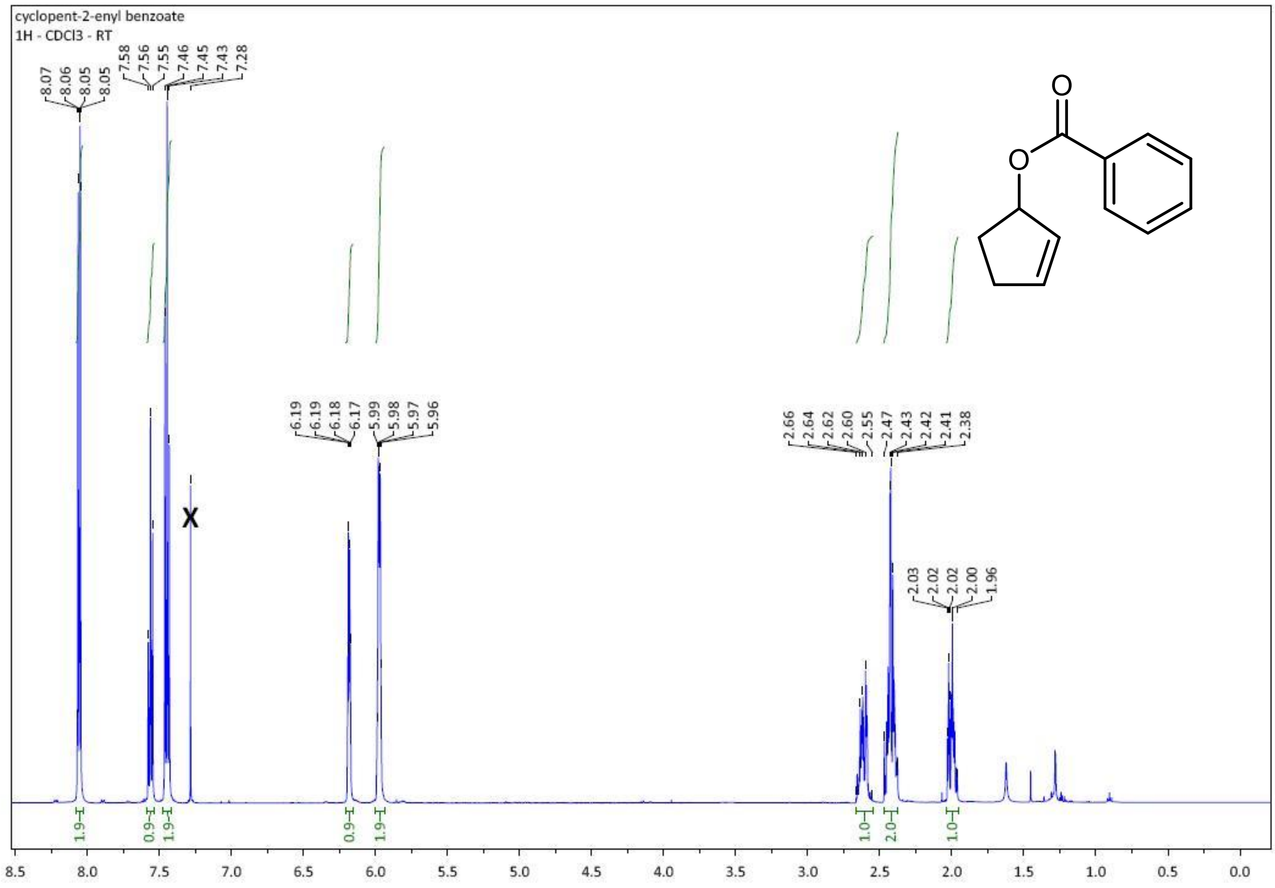

Figure S13. ^{1}H-NMR of compound **5** in $CDCl_3$.

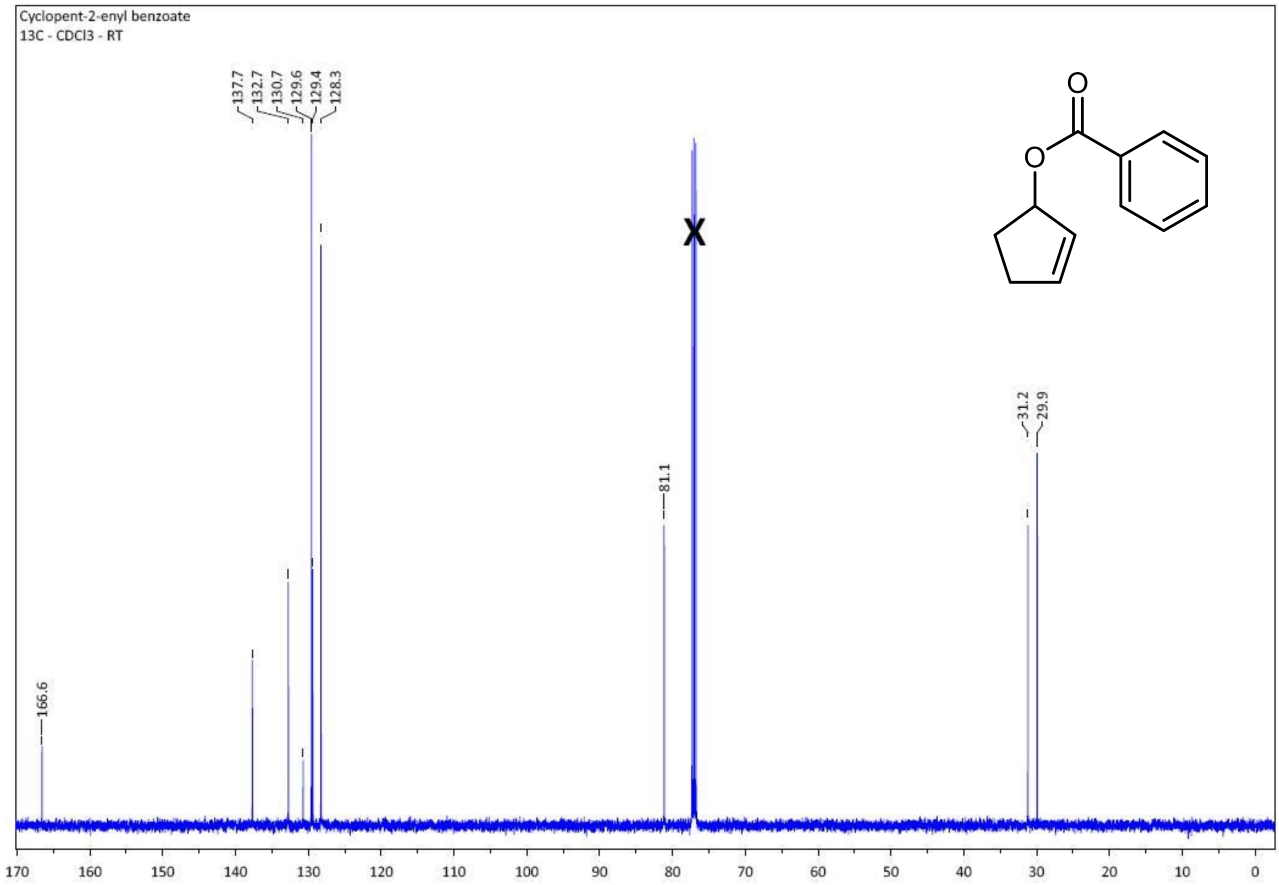

Figure S14. ^{13}C-NMR of compound **5** in $CDCl_3$.

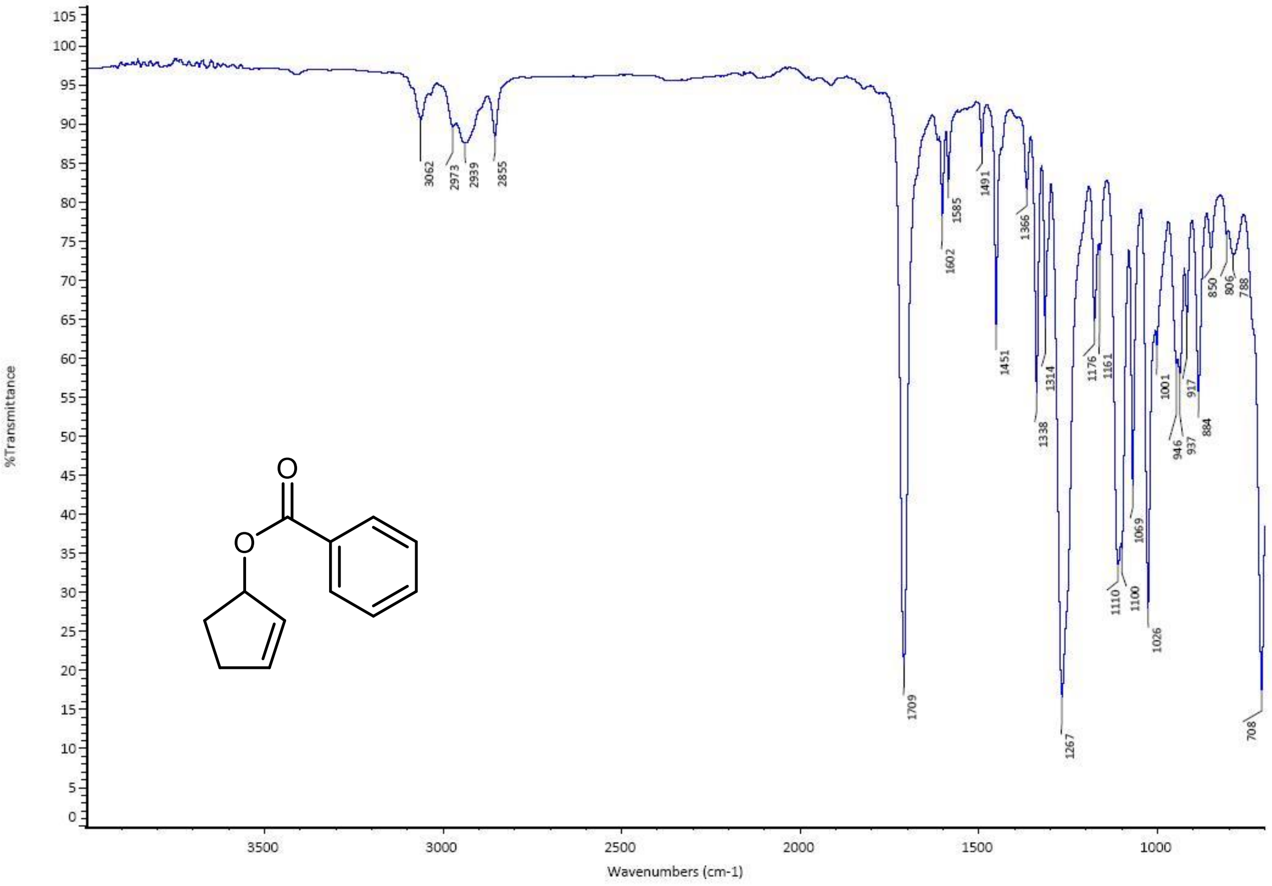

Figure S15. FT-IR spectrum of compound **5**.

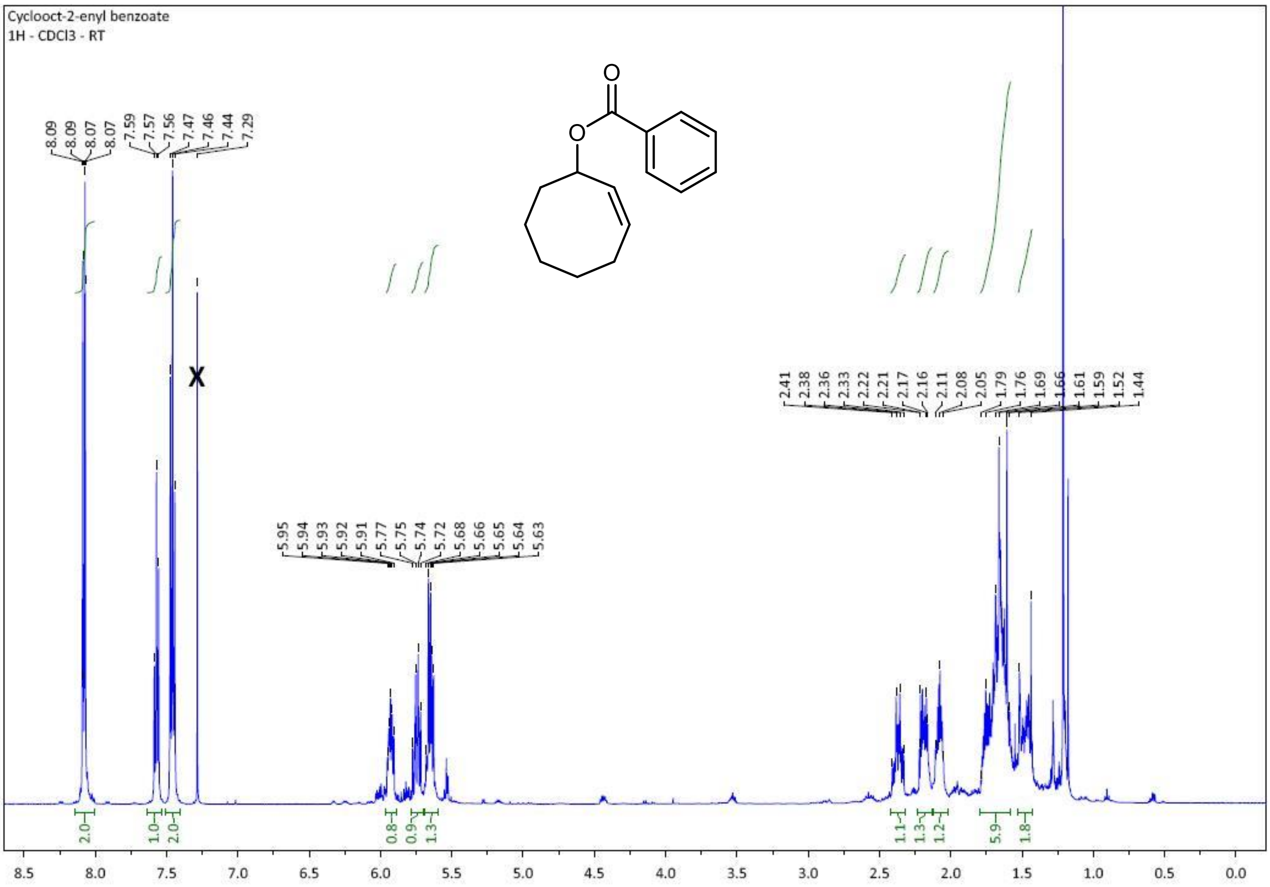

Figure S16. ^{1}H-NMR of compound **6** in $CDCl_3$.

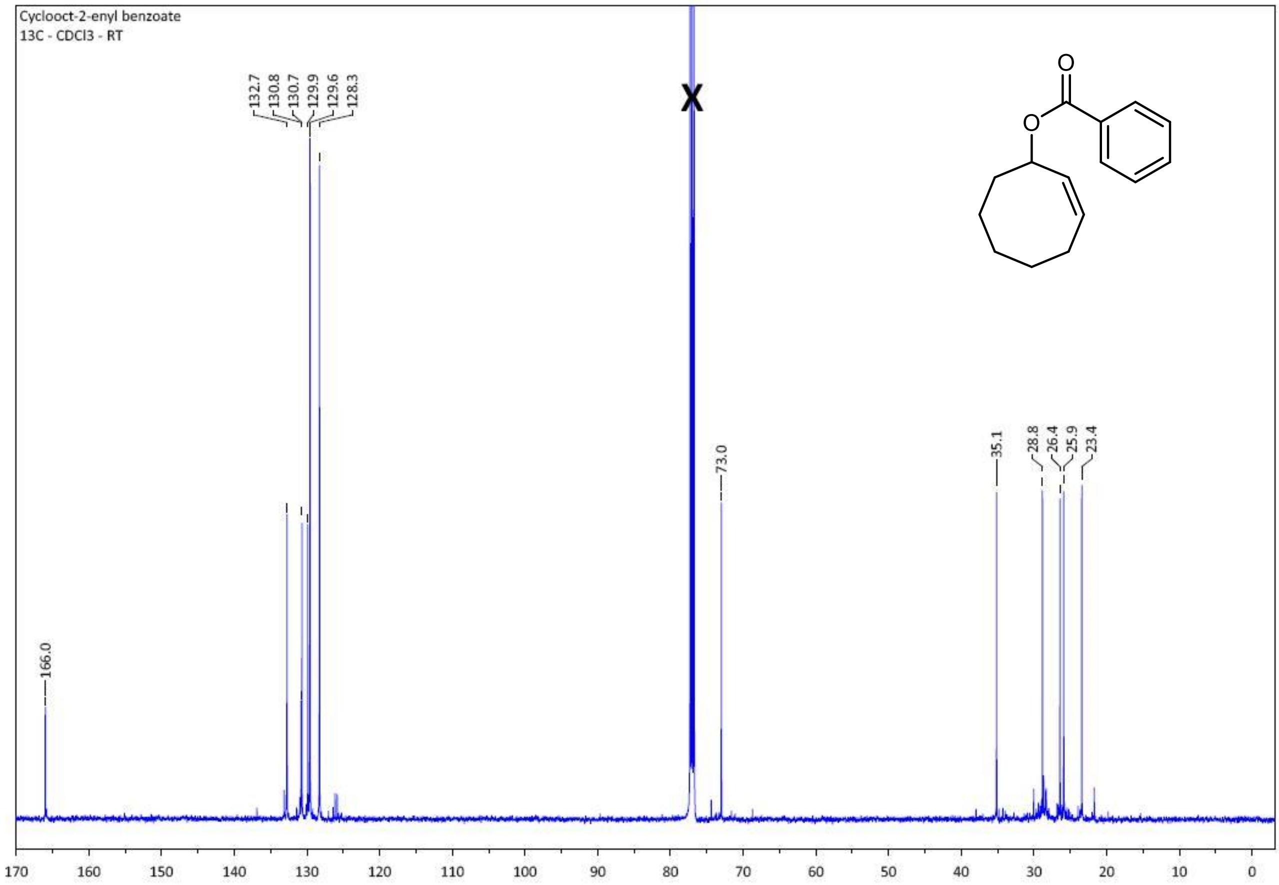

Figure S17. ^{13}C-NMR of compound **6** in $CDCl_3$.

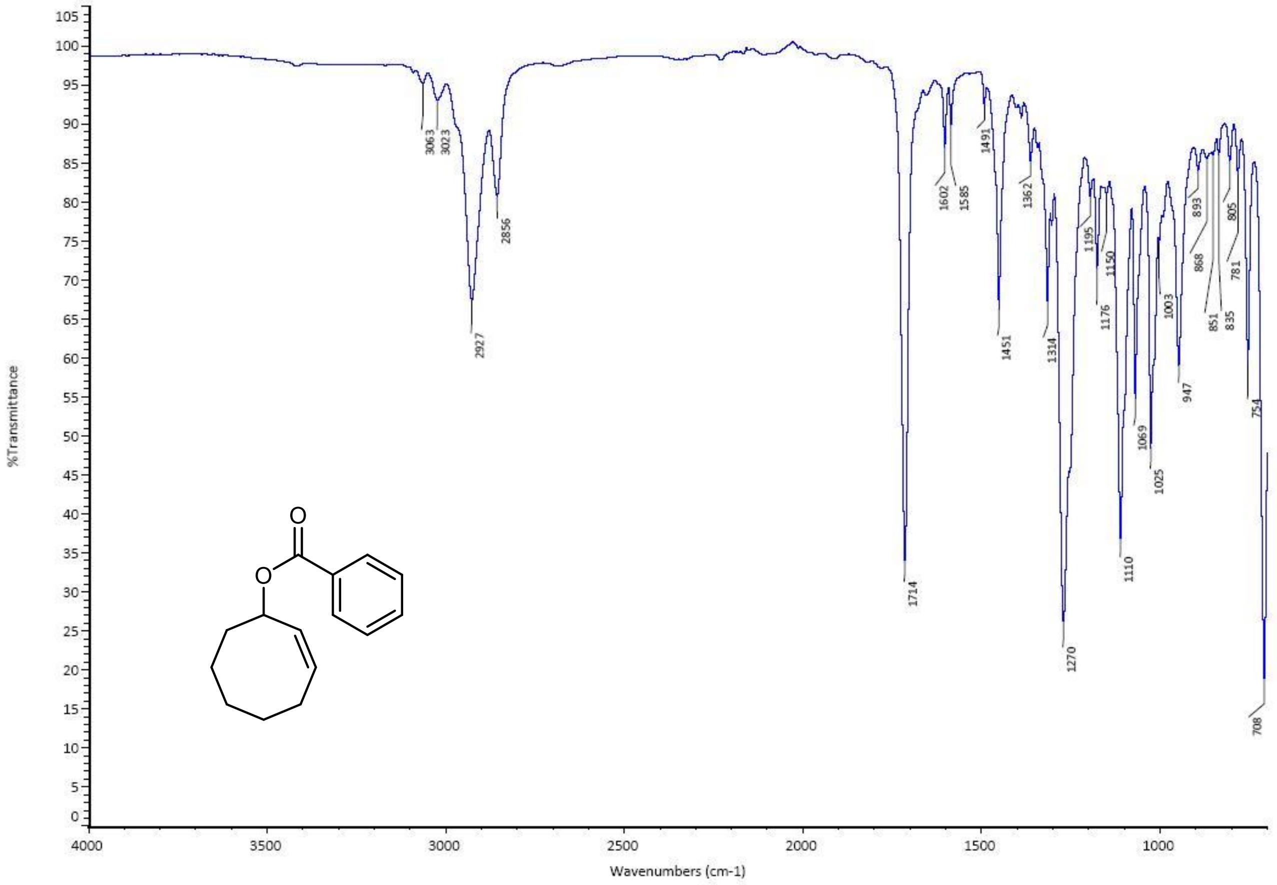

Figure S18. FT-IR spectrum of compound **6**.
